# Supplementary material for: Public health emergency operations centres in Africa: a cross-sectional study assessing the implementation status of core components and areas for improvement, December 2021
Source: BMJ Open. 2023 Jun 20;13(6):e068934. doi: 10.1136/bmjopen-2022-068934 (PMC10314583; doi:10.1136/bmjopen-2022-068934)
Supplement: Author's manuscript [file bmjopen-2022-068934.draft_revisions.pdf]

BMJ Open is committed to open peer review. As part of this commitment we make the peer review history of every article we publish publicly available.

When an article is published we post the peer reviewers' comments and the authors' responses online. We also post the versions of the paper that were used during peer review. These are the versions that the peer review comments apply to.

The versions of the paper that follow are the versions that were submitted during the peer review process. They are not the versions of record or the final published versions. They should not be cited or distributed as the published version of this manuscript.

BMJ Open is an open access journal and the full, final, typeset and author-corrected version of record of the manuscript is available on our site with no access controls, subscription charges or pay-per-view fees (http://bmjopen.bmj.com).

If you have any questions on BMJ Open's open peer review process please email info.bmjopen@bmj.com

BMJ Open

Implementation status of public health emergency operations center in Africa, December 2021

Journal:	*BMJ Open*
Manuscript ID	bmjopen-2022-068934
Article Type:	Original research
Date Submitted by the Author:	07-Oct-2022
Complete List of Authors:	Fekadu, Senait; World Health Organization Regional Office for Africa<br>Gebrewahid, Abrham Lilay; World Health Organization Regional Office for Africa<br>Mankoula, Wessam; African Union, Division of Emergency Preparedness and Response<br>Eteng, Womi; African Union, Division of Emergency Preparedness and Response<br>Lokossou, Virgil; West African Health Organisation, ECOWAS Regional Center for Surveillance and Disease Control<br>Kawe, Yan; World Health Organization Regional Office for Africa<br>Abdullah, Ali; World Health Organisation Regional Office for the Eastern Mediterranean<br>Jian, L; World Health Organization<br>Tut, Mathew; African Union, Division of Emergency Preparedness and Response<br>Wilton, Menchion Chuck; Centers for Disease Control and Prevention<br>Rosenfeld, Emily; Centers for Disease Control and Prevention<br>Bemo, Valerie; Bill & Melinda Gates Foundation<br>Collard, Emily; UK Health Security Agency<br>McGinley, Liz; UK Health Security Agency<br>Halm, Ariane; Robert Koch Institute<br>Aragaw, Merawi; African Union, Division of Emergency Preparedness and Response<br>Conteh, Ishata Nannie; World Health Organization Regional Office for Africa, Emergency Preparedness and Response<br>Braka , Fiona ; World Health Organization Country Office for Nigeria<br>Gueye, Abdou Salam; World Health Organization Regional Office for Africa, Emergency Preparedness and Response Programme
Keywords:	Public health < INFECTIOUS DISEASES, PUBLIC HEALTH, ACCIDENT & EMERGENCY MEDICINE

SCHOLARONE™
Manuscripts

I, the Submitting Author has the right to grant and does grant on behalf of all authors of the Work (as defined in the below author licence), an exclusive licence and/or a non-exclusive licence for contributions from authors who are: i) UK Crown employees; ii) where BMJ has agreed a CC-BY licence shall apply, and/or iii) in accordance with the terms applicable for US Federal Government officers or employees acting as part of their official duties; on a worldwide, perpetual, irrevocable, royalty-free basis to BMJ Publishing Group Ltd ("BMJ") its licensees and where the relevant Journal is co-owned by BMJ to the co-owners of the Journal, to publish the Work in this journal and any other BMJ products and to exploit all rights, as set out in our licence.

The Submitting Author accepts and understands that any supply made under these terms is made by BMJ to the Submitting Author unless you are acting as an employee on behalf of your employer or a postgraduate student of an affiliated institution which is paying any applicable article publishing charge ("APC") for Open Access articles. Where the Submitting Author wishes to make the Work available on an Open Access basis (and intends to pay the relevant APC), the terms of reuse of such Open Access shall be governed by a Creative Commons licence – details of these licences and which Creative Commons licence will apply to this Work are set out in our licence referred to above.

Other than as permitted in any relevant BMJ Author's Self Archiving Policies, I confirm this Work has not been accepted for publication elsewhere, is not being considered for publication elsewhere and does not duplicate material already published. I confirm all authors consent to publication of this Work and authorise the granting of this licence.

For peer review only

Implementation status of public health emergency operations center in Africa, December 2021

[1*]Senait Tekeste Fekadu, [1]Abrham Lilay, [4]Wessam Mankoula, [4]Womi Eteng, [5]Virgil Lokossou, [1]Yan Kawe, [2]Ali Abdullah, [3]Jian L, [4]Mathew Tut, [5]Chuck Menchion Wilton, [5]Emily Rosenfeld, [8]Valerie Bemo, [6]Emily Collard, [6]Liz McGinley, [9]Ariane Halm, [4]Merawi Aragaw, [1]Ishata Conteh, [1]Fiona Braka, [1]Abdu Salam Gueye.

Affiliations

[1]World Health Organization Regional Office for Africa, Brazzaville, Republic of Congo
[2]World Health Organization, Regional Office for Eastern Mediterranean, Cairo, Egypt,
[3]World Health Organization, Headquarters, Geneva, Switzerland,
[4]Africa Centers for Disease Control and Prevention, Division of Emergency Preparedness and Response, Addis Ababa, Ethiopia,
[5]ECOWAS, Regional Center for Surveillance and Disease Control, Abuja, Nigeria
[6]US Centers for Disease Control and Prevention, Atlanta, Georgia, USA,
[7]UK Health Security Agency, Emergency Preparedness, Resilience and Response, London, UK,
[8]Bill and Melinda Gates Foundation, Seattle, Washington, USA.
[9]Robert Koch Institute, Crisis Management Unit, Infectious disease epidemiology Department, Berlin, German and

Email addresses

STF: tekestes@who.int, AL: lilayabrish@yahoo.com, WM: wessamm@africa-union.org, WE: etengw@africa-union.org, VL: vlokossou@wahooas.org, YK: kawey@who.int AA: abdullahal@who.int, JL: ij@who.int, MT: MathewK@africa-union.org, CM: wom0@cdc.gov, EC: emily.collard@ukhsa.gov.uk LM: liz.mcginley@ukhsa.gov.uk, ER: yhc5@cdc.gov, VN: valerie.bemo@gatesfoundation.org, AH: halma@rki.de, MA: merawia@africa-union.org, IC: contehis@who.int, FB: brakaf@who.int, and AG: gueyea@who.int.

Corresponding Author

*Senait Tekeste Fekadu, World Health Organization Regional Office for Africa (WHO AFRO), Congo-Brazzaville, email: tekestes@who.int phone no. +242 06 880 5214

For peer review only

ABSTRACT

Objective: Public health emergencies (PHEs) are still a major problem for African Member States (MS). A functional public health emergency operations center (PHEOC) helps effective emergency management. Sources revealed gaps in establishing functional PHEOCs in the region. Hence, WHO in partnership with key partners conducted a survey in May 2021 to assess the implementation of PHEOCs in Africa.

Method: A self-assessment survey using a standardized tool was done in May 2021 in the African continent and 31 indicators were included to evaluate the status of PHEOC policies and plans, information management, training and exercises, and communication technology infrastructure. Indicators utilizing the PHEOC framework and expert advice were defined and benchmarked at a cumulative score of 80% to ascertain implementation status and fulfillment of minimal requirements for critical emergency management functions.

Results: A total of 51 (93%) MS in Africa responded to the survey. Among these, 41 (80%) MS have established a PHEOC, where only 12 (29%) of them met 80% or more of the minimum requirements needed to perform the critical functions of emergency management. Twenty (49%) and 9 (16%) PHEOCs met 51%-79% and 50% and below the minimum requirements, respectively. More specifically, 10 (24%) and 11 (27%) PHEOCs met 80% or more of the minimum requirements for policies, plans, and procedures and the PHEOC workforce, respectively.

Conclusion: Member States made significant progress in setting up a PHEOC, from nearly zero in 2016 to 41 (80%) in 2021. Of which, one-fifth of the MS have designated a PHEOC meeting at least 80% of the minimum requirements. Significant numbers of MS in the continent either do not have PHEOC or have PHEOCs that met partially the minimal requirements. Hence, this calls for significant collaboration, and joint investment across all stakeholders to establish functional PHEOCs for better emergency operations.

Key Words: Africa, Requirements, Emergency Operations Center, Incident Management System, International Health Regulation, Preparedness, and Response.

SUMMARY

- This is the first article to look in depth at the continent's PHEOC implementation status.
- Most countries established a PHEOC, but with varying capabilities.
- PHEOC was critical in improving the management of various PHEs, including COVID-19.
- WHO and other partners advocate for the establishment of a PHEOC as part of the public health emergency management program.
- This paper can serve as a roadmap for establishing, bolstering, and improving PHEOCs in Africa and other similar contexts.

For peer review only

INTRODUCTION

Public health threats such as disasters and public health emergencies (PHEs) continue to be a major concern for African countries. In Africa, over 100 PHEs are recorded annually, of which more than 80% are caused by infectious diseases[1]. These include emerging and re-emerging diseases, like, Coronavirus Disease 2019 (COVID-19), Monkeypox, Ebola Virus Disease (EVD), Poliomyelitis, Cholera, Rift Valley, Crimean-Congo, and Yellow Fever, among others[2].

Following the adoption of the Integrated Disease Surveillance and Response (IDSR) strategy in 1998 and the International Health Regulations (IHR; 2005), Member States (MS) established systems for surveillance and laboratory to minimize the effects of PHEs on human health, livelihood, trade, and travel by improving preparedness and response initiatives[3-5]. However, recent PHEs revealed huge gaps in capacities. Previous experiences, particularly the EVD outbreak in West Africa from 2014 to 2016, revealed critical gaps in preparing for and responding to public health emergencies. The governments of Guinea, Liberia, and Sierra Leone have acknowledged that poor coordination of efforts and weak health systems hampered the effectiveness of the 2014–2016 Ebola outbreak response[6] (6,). One of the recommendations following the EVD epidemic was to improve capabilities to better prepare and respond to future public health threats. The IHR-2005 requires State Parties to develop, strengthen and maintain their capacity to respond promptly and effectively to PHEs[4].

A functional public health emergency operations center (PHEOC) is an important component in meeting the IHR-2005 minimum requirements and the need to establish a functional PHEOC has been covered as one of the key thematic areas in the joint external evaluation approach developed to help MS assess their capacities to prevent, detect and respond to any public health threats[4]. A PHEOC is a hub for coordination of information and resources where emergency management personnel coordinate preparedness, response, and recovery actions to achieve and maintain adaptability, resilience, and, unity among constituents to effectuate a goal-oriented response to manage the effects or causes of PHEs[7, 8]. The PHEOC employs an Incident Management System (IMS), which is an emergency management structure with sets of procedures and protocols to provide a coordinated approach for all types of emergencies. However, only after a PHEOC has been thoroughly implemented and continuously strengthened can it serve as an effective platform for managing and coordinating information and resources among the multidisciplinary and multisectoral agencies, governments, organizations, and others that prepare for and respond to PHEs.

Following the EVD outbreak in West Africa from 2014 to 2016, the World Health Organization Regional Office for Africa (WHO AFRO), Africa Centers for Disease Control and Prevention (Africa CDC), West African Health Organization (WAHO), and an extensive list of partners heightened the support to MS to establish and operationalize PHEOCs as the top priority. In 2015, WHO published an evidence-based Framework for a Public Health Emergency Operations Center[7]. In the same year, WHO AFRO officially launched a Regional Emergency Operations Center Network (AFR-EOC NET) to collaborate with MS and key partners to assist MS in establishing functional PHEOCs and to promote timely exchange of experiences, best practices, and information[9]. Furthermore, the collaboration between WHO AFRO, Africa CDC, and the United States Centres for Disease Control and Prevention since 2018, is instrumental to the development of an easy-to-use handbook for PHEOC Operations and Management and a PHEOC Legal Framework Guide which provided technical guidance to MS on the development of appropriate policies that authorize PHEOC establishment and full functionality[4, 5].

The PHEOC framework highlights that a fully functional PHEOC is achieved and maintained by putting in place four key components: policies, plans, and procedures; information system and data standard; skilled human resources; and communication technology and physical infrastructure. Africa has made considerable strides in establishing PHEOCs by putting in place these key components to strengthen emergency management. Findings from regional-level meetings on PHEOC implementation status and other exchanges including JEE revealed that there are still gaps in establishing functional PHEOCs in the continent. At present, there is no detailed assessment done on the status of PHEOC establishment and functionality. Therefore, the WHO in partnership with Africa CDC, West African Health

For peer review only - http://bmjopen.bmj.com/site/about/guidelines.xhtml

Organization (WAHO), US CDC, United Kingdom Health Security Agency (UK HSA), Bill and Melinda Gates Foundation (BMGF), and Robert Koch Institute (RKI) conducted a survey to assess the implementation and determine the status of core components required to having functional PHEOCs in the African continent. The findings of this survey will serve as a resource for planning considerations, generate evidence to contextualize minimum and core requirements for functional PHEOCs, and contribute to policy recommendations.

METHODS

Data collection

A standardized self-assessment tool was developed based on Annex 9 of the PHEOC framework to determine the PHEOC minimum requirements. The tool encompasses 31 indicators (**Table 1**) across the PHEOC core components to assess the implementation status and existing capacities. The response to each variable allowed for a yes or no answer and a comment section to provide additional information if any. In May 2021, the survey tool was shared with MS and was administered via an online KoBo Toolbox and MS completed the questionnaire and submitted it online. The online survey questionnaire was shared with 54 MS in Africa (47 of the 54 are MS of the WHO African Region). Data were collected between May – November 2021.

The main question to be answered by this survey was 'How are the PHEOCs performing in meeting the minimum requirements of the core components for functional PHEOCs in the Africa region? To allow for a structure, systematic approach, and comparison, different indicators were selected under each core component of PHEOC and included in the survey to assess the operationalization of PHEOCs across the region.

The survey intended to answer the following questions

- What are the capacities of the PHEOCs in terms of communication technology and physical infrastructure?
- What are the key PHEOC policies, plans, and procedures for emergency management (e.g., legal framework, multi-hazard plan, PHEOC plan/handbook, hazard-specific plans, functional plans, SOPs, etc.) developed and implemented in the PHEOCs?
- What is the strength of the routine and surge emergency management workforce in or accessible to the PHEOCs?
- How is data and information applied for decision-making in the PHEOCs?

Table 1. Indicators assessed in the PHEOC survey by PHEOC core component in Africa

Core components of a PHEOC	***# of indicators assessed***
PHEOC policies, plans, and procedures	12
Human resource, training, and simulation exercise	7
Information management and data standards	4
Communication technology and physical infrastructure	8
Total	31

Data analysis

The minimum PHEOC requirements indicators needed to perform the critical emergency management functions by PHEOCs were determined using the PHEOC framework and consultation from the multi-agency technical expertise supporting PHEOC activities in the continent.

A total of 19 of the 31 indicators (**Table 2**) were prioritized and three levels were used to differentiate the levels of meeting the minimum requirements: 80% and above, 51%-79%, and 50% and below.

Microsoft Excel 2019 was used to analyze the data.

Patient and Public involvement

No patient involved.

For peer review only - http://bmjopen.bmj.com/site/about/guidelines.xhtml

Table 2. List of 19 indicators used to define the fulfillment level of the minimum requirement by PHEOCs in Africa

PHEOC policy, plans, and procedures	Human resource, training, and simulation exercise	Information management and data standards	Communication technology and physical infrastructure
The legal framework authorizes a PHEOC at the national level	PHEOC has the minimum requirements for routine staff (PHEOC manager and key IMS)	A direct link to the national surveillance structure exists	Dedicated PHEOC facility with adequate workstations for the key IMS functions
Legal frameworks include governance structure, core functions, & authority	Staff trained in PHE preparedness and response	Essential data systematically flow to the PHEOC from relevant sectors	Availability of internet access for all workstations and meeting rooms
Relationships, before, during, and after a PHE between stakeholders defined	PHEOC has a comprehensive exercise program involving more than one per year	Able to collect and manage operational information to inform leadership	Availability of electricity with backup power
A policy group to provide strategic guidance established	Staff are routinely trained on guides, plans, and through simulation to validate competencies	PHEOC uses digital solutions to process its information	
Handbook for PHEOC operations and management is in place	Could contact a roster of trained personnel to fill IMS positions when PHEOC activated		
The multi-hazard response plan is approved			
A business continuity plan (BCP) is in place			

RESULTS

A total of 51 (93%) MS in the African continent responded to the survey. Forty-one (80%) MS responded that they have established a PHEOC at the national level and 21 MS had a PHEOC at the sub-national level. Within these, 41 of the 47 MS in the WHO African Region responded and 36 MS had a national PHEOC, whereas 17 MS were at the sub-national level.

Table 3 demonstrates that 12 (29%) of the 41 PHEOCs in the continent and 11 (31%) PHEOCs in the AFRO region met 80% or more of the minimum requirements criteria for the four PHEOC core components. Twenty (49%) of the PHEOCs in the continent met 51%-79% and 9 (16%) met 50% and below the minimum requirements respectively. The number of PHEOCs in the continent that meet the criteria for the four PHEOC core components are displayed in **Table 3**.

For peer review only - http://bmjopen.bmj.com/site/about/guidelines.xhtml

Table 3. Member States minimum requirements criteria fulfillment by PHEOC core component in Africa, 2021

Core components of a PHEOC	*Met 80% and above # (%)*	*Met 51%-79% # (%)*	*Met 50% and below # (%)*
PHEOC policies, plans, and procedures	10 (24)	17 (41)	14 (34)
Human resource, training, and simulation exercise	11 (27)	12 (29)	17 (41)
Information management and data standards	24 (59)	10 (24)	7 (17)
Communication technology and physical infrastructure	18 (44)	11 (27)	12 (29)
Total (n=41)	**12 (29)**	**20 (49)**	**9 (22)**

Core component 1: PHEOC policies, plans, and procedures

Twenty-five (61%) of the PHEOCs have already developed and enacted a legal framework to establish a PHEOC and 23 (56%) of the PHEOC legal frameworks have covered governance structure, core functions, and the scope of authority (**Figure 1**). In addition, twenty-nine (71%) of the PHEOCs had a policy group to provide strategic guidance for the PHEOC operationalization.

Twenty (49%) of the PHEOCs implemented a handbook for PHEOC operations and management to guide their day-to-day operations. Twenty-five (61%) have developed a national multi-hazard response plan that includes the concept of operations addressing priority risks and 19 (46%) of them were endorsed. In 24 (59%) of the PHEOCs, a response plan covering various stakeholders' roles and responsibilities supporting response efforts was in place. Thirty-one (76%) of the PHEOCs had IMS or comparable response structure and 25 (61%) of the PHEOCs could activate within 120 minutes as per JEE recommendations (**Table 4**).

In the WHO African Region, 18 (44%) MS had a handbook for PHEOC operations and management. Besides, 17 (41%) and 23(56%) MS had an approved multi-hazard response plan and the capacity to activate within 120 minutes, respectively.

Table 4. PHEOCs with the capacity for PHEOC plans and procedures, core component 1 (PHEOC policies, plans, and procedures) in Africa, 2021

PHEOC plans and procedures	*Number (#)*	*Of the PHEOCs (%)*
Operational IMS or comparable structure in place	31	76
Multi-hazard response plan including the concept of operations that have been developed	25	61
Could activate the PHEOC within 120 minutes after the decision made	25	61
A response plan that covers the roles and responsibilities of orgs. supporting the response is in place	24	59
Business Continuity Plan (BCP) is in place	23	56
PHEOCs that implemented handbook for operations and management	20	49
Multi-hazard response plan including the concept of operations that have been approved	19	46

For peer review only - http://bmjopen.bmj.com/site/about/guidelines.xhtml

Core component 2: Human resource, training, and simulation exercise

Thirty-eight (93%) of the PHEOCs indicated that at least one manager or focal point was present to oversee day-to-day operations and management of the PHEOCs and 26 (63%) had the minimum expected personnel: PHEOC manager, lead for operations, planning, logistics, finance and administration, communications officer, and ICT expert) to carry out routine preparedness activities. In 17 (41%) PHEOCs, staff were frequently oriented on PHEOC guides, and emergency management, and 14 (34%) PHEOCs regularly simulated exercises (SIMEX) to test different capacities. When the IMS was activated, 32 (78%) PHEOCs were able to contact a roster of pre-trained experts to assist with response coordination activities (**Table 5**).

In the WHO African Region, 21 (51%) PHEOCs had the minimum expected routine staff, and 29 (71%) PHEOCs had staff trained on PHE preparedness and response.

Table 5. PHEOCs with the capacity for core component 2 (human resources and simulation exercises) in Africa, 2021

Parameters under human resource, training, and simulation exercise	*Number (#)*	*Of the PHEOCs (%)*
PHEOCs with a designated PHEOC Manager	38	93
Trained staff on PHE preparedness and response	33	80
PHEOCs could contact a roster of trained personnel	32	78
PHEOCs with personnel to run response functions 24/7	26	63
*PHEOCs with the minimum routine staff for IMS functions**	26	63
PHEOCs with a dedicated training program	21	51
Routinely trained staff on existing PHEOC guidance documents and conducted exercises to validate competencies	17	41
PHEOCs with more than one exercise program per year and produces after-action reports	14	34

NB. *PHEOC manager and leads for OPs, Planning, Logistics, Finance and admin, Comms. officer, & ICT manager.

Core component 3: Information management and data standards

There was a digital solution to process the data and information acquired from various sources in 33 (80%) of the PHEOCs. Data were routinely coming from relevant sectors (e.g., surveillance and/or local health systems) in 32 (78%) PHEOCs. Thirty-five (85%) PHEOCs could collect and manage operational information (situational awareness) to inform action whereas 26 (63%) PHEOCs were producing visual dashboards to convey a concise picture of the response and situation.

Core component 4: Communication technology and physical infrastructure

As indicated in **Figure 2**, 27 (66%) of the PHEOCs had sufficient computer workstations, whereas 33 (80%) had workstations capable of serving the essential IMS personnel. Twenty-six (63%) had internet connectivity for the workstations and meeting rooms and 27 (66%) PHEOCs could hold a teleconference.

Twenty-eight (68%) and 21 (51%) PHEOCs with space for at least the IMS staff and adequate internet access, respectively, were from MS of the WHO African Region.

DISCUSSION

Managing adverse health outcomes and economic disruption attributed to PHEs requires effective preparedness and response procedures[2, 10]. A public health emergency operations center (PHEOC) is a hub designed to effectively coordinate information and resources during the management of PHEs. Thoroughly implemented and continuously strengthened PHEOCs that can support crisis management are part of the core capacities required under the IHR-2005[10]. A public health emergency operations

For peer review only - http://bmjopen.bmj.com/site/about/guidelines.xhtml

center (PHEOC) is a hub designed to effectively support the coordination of information and resources during the management of PHEs. To function efficiently, the PHEOC must have appropriate policies, plans, and procedures in place, as well as dependable and effective information management systems, communication technology, physical infrastructure, and well-trained staff; and it must be exercised regularly[7]. The authors present the status of implementation of PHEOCs in Africa and recommend strategies for the Member States to consider for accelerating this meaningful effort.

According to the findings of the survey, MS made significant progress in setting up PHEOC to strengthen their emergency management capability, from nearly zero in 2016 to 41 (80%) in 2021. The results, however, showed that MS are at different levels of implementing the PHEOC core components and only 12 (29%) of the PHEOCs in the region met at least 80% of the minimum requirements. Furthermore, 20 (49%) and 9 (16%) of the PHEOCs in the continent met 51%-79% and 50% and below the minimum requirements respectively. The capacity related to the basic indicators, however, differed. For example, in most of the MS, the core components of PHEOC policies, plans, and procedures and the PHEOC workforce appear to have a critical gap, where only 10 (24%) and 11 (27%) met 80% and above of the minimum requirements. The main reason for this could be technical capacity, financial or other constraints. Most of the MS, on the other hand, had the required capability in terms of core component 3, Information management, and data standards. This emphasizes the importance of providing technical assistance to support the development of key PHEOC documents and training for routine and surge staff to MS with limited capacities.

A legal framework for approving the PHEOC's establishment and operation, a handbook for its operations and management, event or hazard-specific response and management plans, an incident action plan, and other pertinent plans and procedures are key components of the PHEOC and must ideally be in place before any public health emergency occurs[7]. Among the key indicators under the core component of PHEOC policies, plans, and procedures, a gap was observed in most MS in having a handbook for PHEOC operations and management (20 MS only), a multi-hazard response plan including the concept of operations that have been approved only in 20 MS and a Business Continuity Plan (BCP) was in place in 23 MS. This might affect the effectiveness of the PHEOC response during emergencies due to the absence of clear guidance and operational response plans including the contingency plan.

Regarding the workforce capacity, despite most of the MS meeting the key requirements in some of the parameters, a gap was observed in most of the MS regarding a dedicated training program, limited routinely trained staff on existing PHEOC guidance documents, and very few exercises to validate competencies were conducted (41% of MS only). PHEOCs with more than one exercise program per year and producing after-action reports constitute a key gap observed in this assessment (34% of MS only).

The personnel needed for a PHEOC may vary by many factors, including the emergency's size and complexity. However, a PHEOC usually has a certain number of full-time staff responsible for routine operations of the PHEOC while a scalable number of surge personnel will be needed when the PHEOC is activated. The surge personnel, therefore, should be identified and trained in IMS and specific functions in advance of the emergencies and an up-to-date roster should be maintained[11]. When the PHEOC is activated, it should be staffed with a team of subject matter experts, analysts, logistics staff, and support staff[4]. The function and staffing of the EOC should be assessed through an ongoing series of training and exercises[4, 12]. Well-trained professionals are the key to the operations of PHEOC and trained experts who know what to do are critical to building a functioning PHEOC[11, 13, 14]. Training should be purposefully conducted according to the capabilities required by the PHEOC. To increase the number of competent and skilled health workers for public health emergencies, interventions targeted at improving their knowledge and skills are essential[15]. Exercises should be carried out to simulate the emergency scenario to familiarize them with their roles and responsibilities and for this discussion-based and operations-based exercises are usually used[8].

An EOC's lifeblood is information. Information management is a critical component of a functional PHEOC, and it entails gathering, analyzing, and distributing data promptly[8-12, 16]. Event-specific data, event management information, and context data are the three categories of data that must be

For peer review only - http://bmjopen.bmj.com/site/about/guidelines.xhtml

consistently recorded, analyzed, and displayed in a PHEOC[7]. During response coordination, PHEOC requires specific types of data depending on the type of PHE. Data should be gathered according to local conditions. During activation, EOCs use data technologies and informal networks of public health professionals to monitor epidemiologic data and field reports from several sources[13]. In addition, the PHEOC should maintain clear and updated information about the incident or disaster. Effective communication is mandatory to keep the public trust in the messages and the function of the PHEOC[17].

Among the indicators under the core component of communication technology and physical infrastructure, a critical gap was observed in having interoperable radio communication and other logistic facilities. However, most MS performed better in other indicators under this core component. The primary reason for the gaps observed might be limitations in internet accessibility, financial limitations, and a limitation in technical capabilities. This primarily affected the effectiveness of the PHEOC functionality in terms of easy communication, especially at the sub-national level. Furthermore, this assessment finding demonstrated that most of the MS have a PHEOC capacity only at the national level. There is still a critical gap at the sub-national level where most emergencies are immediately responded to. Therefore, addressing such gaps might be critically considered in MS with lower capacity. The PHEOC should have the potential to acquire the technologies such as computers, phones, TV plasma screens, projectors, and radio systems that support telecommunications, information management, and visualization of operational information[18]. This supports the PHEOC in facilitating communication and information management, resulting in more effective response coordination.

LIMITATION

The survey was self-administered hence respondents were likely not to adjudge performance status on the same scale. The largely binary nature of the questionnaire (yes/no options) may have limited the documentation of minor but important progress made.

CONCLUSION

The African Member States made significant progress in setting up a public health EOC to improve the emergency management capabilities, where from nearly zero in 2016 to 41 (80%) MS put in place in 2021. Of these, one-fifth (12 MS) designated a PHEOC that met at least 80% of the minimum at the national level. In addition, 20 (49%) and 9 (16%) MS have PHEOCs that met 51%-79% and 50% and below the minimum requirements respectively. However, the PHEOCs in many MS are of different capabilities and meet only some of the requirements and some MS still have no PHEOCs.

The main bottlenecks for implementing functional PHEOC in Africa include the absence of a legal framework that clearly defines its mandate, the lack of a standing policy group to provide operational support and strategic direction, unapproved plans and procedures, and limited human resources and funding for operations and sustainability.

Therefore, it is crucial to enhance the implementation of functional PHEOC according to the WHO framework and accompanying handbooks and guides. The implementation planning processes require the involvement of all relevant stakeholders. Besides, regional, and international partners need to support MS' efforts to address these gaps in developing functional PHEOC.

Contributors

ST, WM, WE, VK, YK, AA, JL, MT, CM, ER, VB, EC, LM, AH, MA, IC, FB, and AG prepared the survey tool. ST and AL conducted relevant article searches, developed a data analysis plan, analyzed the data, and wrote the manuscript. ST, AL, WM, WE, VK, YK, AA, JL, MT, CM, ER, VB, EC, LM, AH, MA, IC, FB, and AG contributed to the writing and reviewed the final manuscript. All authors read and approved the final manuscript.

Competing interests

None declared.

Funding statement

There is neither a financial relationship nor sponsorship with any organization to be declared.

For peer review only - http://bmjopen.bmj.com/site/about/guidelines.xhtml

Ethical approval
The survey was conducted in line with the organization's policies and regulations.

Availability of data and materials
The datasets from the survey used in this article are available upon reasonable request.

Acknowledgment
We thank all the MS in the continent for their significant contributions to the survey. We also want to thank everyone who helped review the survey tool and article.

REFERENCES
1. Health S, Centre O. Afro shoc 1. 2014. 1–67 p.
2. Talisuna AO, Okiro EA, Yahaya AA, Stephen M, Bonkoungou B, Musa EO, et al. Spatial and temporal distribution of infectious disease epidemics, disasters, and other potential public health emergencies in the World Health Organisation Africa region, 2016-2018. Global Health. 2020;16(1):1–13.
3. WHO. This booklet introduces all 11 sections of the Integrated Disease Surveillance and Response (IDSR) Technical Guidelines. 2019;
4. Kamradt-Scott A. The International Health Regulations (2005). Int Organ Law Rev. 2019;16(2):242–71.
5. WHO. Public health events of initially unknown etiology : A framework for preparedness and response in the African Region. 2014; Available from: http;//www.who.int/iris/handle/10665/112832
6. Agbo S, Gbaguidi L, Biliyar C, Sylla S, Fahnbulleh M, Dogba J, et al. Establishing National Multisectoral Coordination and collaboration mechanisms to prevent, detect, and respond to public health threats in Guinea, Liberia, and Sierra Leone 2016–2018. One Heal Outlook. 2019;1(1):1–13.
7. WHO. Framework for a Public Health Emergency Operations Centre. MMWR Morb Mortal Wkly Rep [Internet]. 2015;50(RR13)(July 27):1–35. Available from: www.who.int/about/licensing/%0Ahttps://apps.who.int/iris/bitstream/handle/10665/196135/9789241565134_eng.pdf?sequence=1
8. WHO; A Systematic Review of Public Health Emergency Operations Centres (EOC). 2013;(December):11.
9. Network of Public Health Emergency Operation Centers established to protect lives _ WHO _ Regional Office for Africa.
10. World Health Organization. Strengthening health-system emergency preparedness. 2012;96. Available from: http://www.euro.who.int/__data/assets/pdf_file/0008/157886/e96187.pdf
11. Xu M, Li SX. Analysis of good practice of public health Emergency Operations Centers. Asian Pac J Trop Med [Internet]. 2015;8(8):677–82. Available from: http://dx.doi.org/10.1016/j.apjtm.2015.07.006
12. FEMA. National Incident Management System 2017 learning materials. 2017;
13. Balajee SA, Pasi OG, Etoundi AGM, Rzeszotarski P, Do TT, Hennessee I, et al. Sustainable model for public health emergency operations centers for global settings. Emerg Infect Dis. 2017;23(December): S190–5.
14. CDC. CDC Emergency Operations Center | CDC. CDC Emergency Operations Center | CDC. 2020.
15. Olu O, Usman A, Kalambay K, Anyangwe S, Voyi K, Orach CG, et al. What should the African health workforce know about disasters? Proposed competencies for strengthening public health disaster risk management education in Africa. BMC Med Educ. 2018;18(1).
16. Scanlon J. The Role of EOCs in Emergency Management: A Comparison of American and Canadian Experience. Vol. 12, International Journal of Mass Emergencies and Disasters. 1994. p. 51–75.
17. Rebmann T, Carrico R, English JF. Lessons public health professionals learned from past

For peer review only - http://bmjopen.bmj.com/site/about/guidelines.xhtml

disasters. Public Health Nurs. 2008;25(4):344–52.

18. Simon R, Teperman S. WTC Lessons for disaster management. 2001;5(6):5–7.

For peer review only

List of Figures

Figure 1. PHEOCs with the capacity for PHEOC legal frameworks, core component 1 (PHEOC policies, plans, and procedures) in Africa, 2021

Figure 2. PHEOCs with the capacity for core component 4 (communication technology and physical infrastructure) minimum requirements in Africa, 2021

For peer review only

For peer review only - http://bmjopen.bmj.com/site/about/guidelines.xhtml

Figure 1. PHEOCs with the capacity for PHEOC legal frameworks, core component 1 (PHEOC policies, plans, and procedures) in Africa, 2021

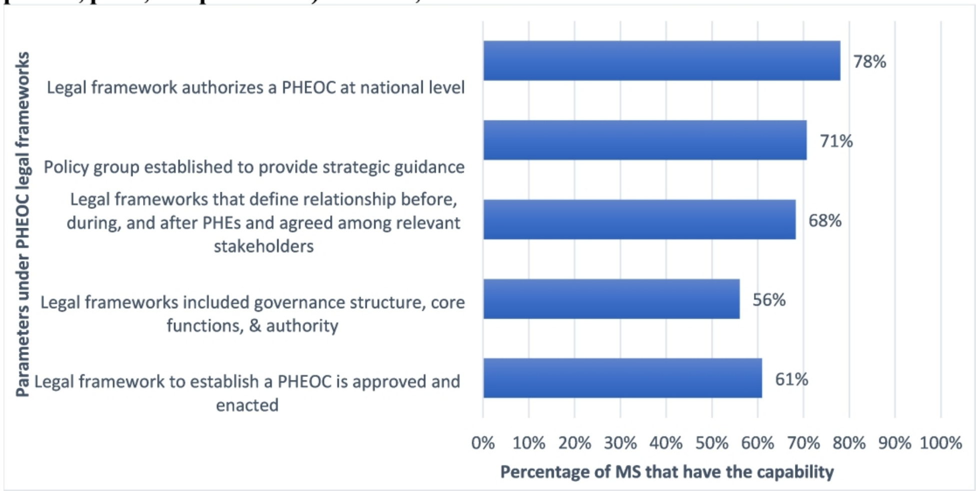

165x92mm (600 x 600 DPI)

Figure 2. PHEOCs with the capacity for core component 4 (communication technology and physical infrastructure) minimum requirements in Africa, 2021

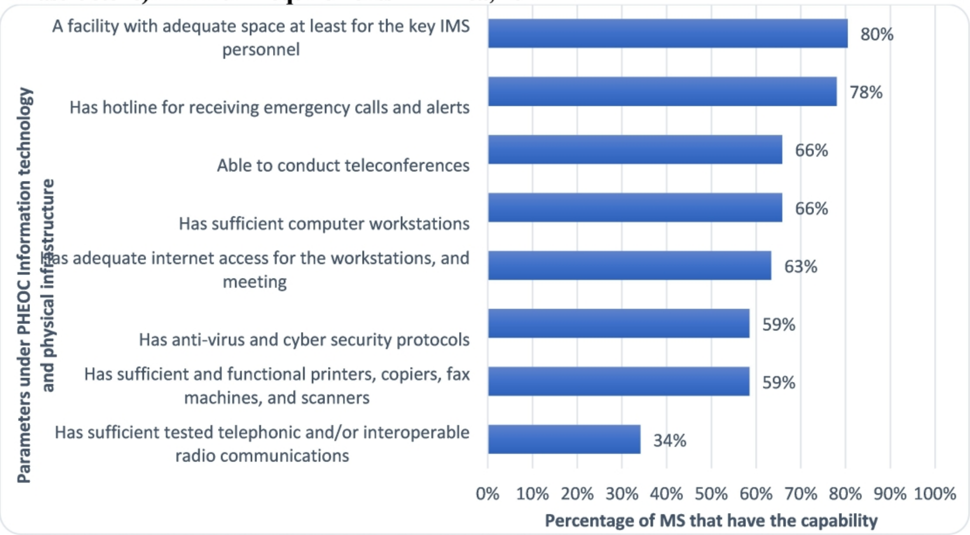

165x100mm (600 x 600 DPI)

BMJ Open

Public health emergency operations centres in Africa: implementation status of core components and areas for improvement, December 2021

Journal:	*BMJ Open*
Manuscript ID	bmjopen-2022-068934.R1
Article Type:	Original research
Date Submitted by the Author:	04-Jan-2023

Complete List of Authors:	Fekadu, Senait; World Health Organization Regional Office for Africa<br>Gebrewahid, Abrham Lilay; World Health Organization Regional Office for Africa<br>Mankoula, Wessam; African Union, Division of Emergency Preparedness and Response<br>Eteng, Womi; African Union, Division of Emergency Preparedness and Response<br>Lokossou, Virgil; West African Health Organisation, ECOWAS Regional Center for Surveillance and Disease Control<br>Kawe, Yan; World Health Organization Regional Office for Africa<br>Abdullah, Ali; World Health Organisation Regional Office for the Eastern Mediterranean<br>Jian, L; World Health Organization<br>Tut, Mathew; African Union, Division of Emergency Preparedness and Response<br>Wilton, Menchion Chuck; Centers for Disease Control and Prevention<br>Rosenfeld, Emily; Centers for Disease Control and Prevention<br>Bemo, Valerie; Bill & Melinda Gates Foundation<br>Collard, Emily; UK Health Security Agency<br>McGinley, Liz; UK Health Security Agency<br>Halm, Ariane; Robert Koch Institute<br>Aragaw, Merawi; African Union, Division of Emergency Preparedness and Response<br>Conteh, Ishata Nannie; World Health Organization Regional Office for Africa, Emergency Preparedness and Response<br>Braka , Fiona ; World Health Organization Country Office for Nigeria<br>Gueye, Abdou Salam; World Health Organization Regional Office for Africa, Emergency Preparedness and Response Programme

**Primary Subject Heading**:	Public health
Secondary Subject Heading:	Public health, Health policy
Keywords:	PUBLIC HEALTH, ACCIDENT & EMERGENCY MEDICINE, Health policy < HEALTH SERVICES ADMINISTRATION & MANAGEMENT

SCHOLARONE™
Manuscripts

I, the Submitting Author has the right to grant and does grant on behalf of all authors of the Work (as defined in the below author licence), an exclusive licence and/or a non-exclusive licence for contributions from authors who are: i) UK Crown employees; ii) where BMJ has agreed a CC-BY licence shall apply, and/or iii) in accordance with the terms applicable for US Federal Government officers or employees acting as part of their official duties; on a worldwide, perpetual, irrevocable, royalty-free basis to BMJ Publishing Group Ltd ("BMJ") its licensees and where the relevant Journal is co-owned by BMJ to the co-owners of the Journal, to publish the Work in this journal and any other BMJ products and to exploit all rights, as set out in our licence.

The Submitting Author accepts and understands that any supply made under these terms is made by BMJ to the Submitting Author unless you are acting as an employee on behalf of your employer or a postgraduate student of an affiliated institution which is paying any applicable article publishing charge ("APC") for Open Access articles. Where the Submitting Author wishes to make the Work available on an Open Access basis (and intends to pay the relevant APC), the terms of reuse of such Open Access shall be governed by a Creative Commons licence – details of these licences and which Creative Commons licence will apply to this Work are set out in our licence referred to above.

Other than as permitted in any relevant BMJ Author's Self Archiving Policies, I confirm this Work has not been accepted for publication elsewhere, is not being considered for publication elsewhere and does not duplicate material already published. I confirm all authors consent to publication of this Work and authorise the granting of this licence.

For peer review only

BMJ Open

Public health emergency operations centres in Africa: implementation status of core components and areas for improvement, December 2021

[1*]Fekadu Senait Tekeste, [1]Abrham Lilay, [4]Wessam Mankoula, [4]Womi Eteng, [5]Lokossou Virgil, [1]Yan Kawe, [2]Ali Abdullah, [3]Jian L, [4]Mathew Tut, [5]Chuck Menchion Wilton, [5]Emily Rosenfeld, [8]Valerie Bemo, [6]Emily Collard, [6]Liz McGinley, [9]Ariane Halm, [4]Merawi Aragaw, [1]Conteh Ishata Nannie, [1]Braka Fiona, [1]Gueye Abdou Salam.

Affiliations

[1]World Health Organization Regional Office for Africa, Brazzaville, Republic of Congo
[2]World Health Organization, Regional Office for Eastern Mediterranean, Cairo, Egypt,
[3]World Health Organization, Headquarters, Geneva, Switzerland,
[4]Africa Centres for Disease Control and Prevention, Division of Emergency Preparedness and Response, Addis Ababa, Ethiopia,
[5]ECOWAS, Regional Center for Surveillance and Disease Control, Abuja, Nigeria
[6]US Centers for Disease Control and Prevention, Atlanta, Georgia, USA,
[7]UK Health Security Agency, Emergency Preparedness, Resilience and Response, London, UK,
[8]Bill and Melinda Gates Foundation, Seattle, Washington, USA.
[9]Robert Koch Institute, Crisis Management Unit, Infectious disease epidemiology Department, Berlin, German and

Email addresses

STF: tekestes@who.int, AL: lilayabrish@yahoo.com, WM: wessamm@africa-union.org, WE: etengw@africa-union.org, VL: vlokossou@wahooas.org, YK: kawey@who.int AA: abdullahal@who.int, JL: ij@who.int, MT: MathewK@africa-union.org, CM: wom0@cdc.gov, EC: emily.collard@ukhsa.gov.uk LM: liz.mcginley@ukhsa.gov.uk, ER: yhc5@cdc.gov, VN: valerie.bemo@gatesfoundation.org, AH: halma@rki.de, MA: merawia@africa-union.org, IC: contehis@who.int, FB: brakaf@who.int, and AG: gueyea@who.int.

Corresponding Author

*Senait Tekeste Fekadu, World Health Organization Regional Office for Africa (WHO AFRO), Congo-Brazzaville, email: tekestes@who.int phone no. +242 06 880 5214

For peer review only

ABSTRACT

Objective The survey was conducted to assess implementation status of national public health emergency operations centres (PHEOCs) in all countries in Africa.

Design Cross-sectional study.

Setting 54 national PHEOC focal points from 54 countries in Africa, between May to November 2021. Variables needed to assess PHEOC capacities included, and descriptive analysis was done.

Results A total of 51 (93%) MS in Africa responded to the survey. Among these, 41 (80%) MS have established a PHEOC and 12 (29%) of them that met 80% or more of the minimum capabilities needed to perform the critical emergency management functions were labelled as category I. Twenty (49%) and 9 (16%) PHEOCs that met 51%-79% and 50% and below the minimum capabilities were labelled as category II and III, respectively. More specifically, 10 (24%) and 11 (27%) PHEOCs met 80% or more of the minimum capabilities for policies, plans, and procedures and the PHEOC workforce, respectively.

Conclusions Member States made considerable progress in setting up a PHEOC. Since the commencement of the initiative in 2015, 41 (80%) MS established a PHEOC in 2021. Of which, one-fifth (12) of the MS have designated a PHEOC meeting at least 80% of the minimum capabilities to operate the critical emergency functions. There are still MS in the continent either do not have PHEOC or have PHEOCs that met partially the minimal capabilities. Hence, this calls for significant collaboration, and joint investment across all stakeholders to establish functional PHEOCs for better emergency operations.

Strengths and limitations

- This is the first article to look in depth at the implementation status of PHEOC in the African continent.
- This article could serve as a resource for planning considerations to support countries in the continent.
- Qualitative study should be done to account the progress made across the core capabilities.

For peer review only - http://bmjopen.bmj.com/site/about/guidelines.xhtml

INTRODUCTION

Public health threats such as disasters and public health emergencies (PHEs) continue to be a major concern for African countries. In Africa, over 100 PHEs are recorded annually, of which more than 80% are caused by infectious diseases[1]. These include emerging and re-emerging diseases, like, coronavirus disease 2019 (COVID-19), monkeypox, Ebola virus disease (EVD), polio, cholera, Rift Valley fever, Crimean-Congo hemorrhagic fever, and yellow fever, among others[2].

Since the adoption of the International Health Regulations (IHR) 2005, Member States (MS) established systems to minimize the effects of PHEs by improving preparedness and response capabilities[3-5]. However, recent PHEs revealed huge gaps in capacities. Previous experiences, particularly the EVD outbreak in West Africa from 2014 to 2016, revealed critical gaps in preparing for and responding to PHEs. The governments of Guinea, Liberia, and Sierra Leone have acknowledged that poor coordination of efforts and weak health systems hampered the effectiveness of the 2014–2016 EVD outbreak response[6]. Improving preparedness and response capacities of MS as required by IHR (2005) which requires State Parties to develop, strengthen and maintain their capacity to respond effectively to PHEs was one of the recommendations that has been made following the EVD outbreak [6].

A functional public health emergency operations center (PHEOC) is an important component in meeting the IHR (2005) minimum capabilities and the need to establish a functional PHEOC has been covered as one of the key thematic areas in the joint external evaluation (JEE) developed to help MS assess their capacities to prevent, detect and respond to any public health threats[4]. A PHEOC is a hub for effective coordination of information and resources during the management of PHEs[7, 8]. The PHEOC employs an incident management system (IMS), an emergency management structure with sets of procedures and protocols to provide a coordinated approach for all types of PHEs. However, only after a PHEOC has been thoroughly implemented and continuously strengthened can it serve as an effective platform for managing and coordinating information and resources among the multidisciplinary and multisectoral agencies, governments, organizations, and others that prepare for and respond to PHEs.

Post the 2014 to 2016 EVD outbreak in West Africa, World Health Organization Regional Office for Africa (WHO AFRO), Africa Centres for Disease Control and Prevention (Africa CDC), West African Health Organization (WAHO), and other partners heightened the support to MS to establish and operationalize PHEOCs as the top priority. As part of the support, WHO published an evidence-based Framework for a Public Health Emergency Operations Center in 2015[7]. In the same year, WHO AFRO officially launched a regional emergency operations center network (AFR-EOCNET) to collaborate with MS and key partners to assist MS in establishing functional PHEOCs and to promote timely exchange of experiences, best practices, and information[9]. Furthermore, the collaboration between WHO AFRO, Africa CDC, and United States Centers for Disease Control and Prevention (US CDC) since 2018, is instrumental to the development of an easy-to-use handbook for PHEOC operations and management and a PHEOC legal framework guide which provided technical guidance to MS on the development of appropriate policies that authorize PHEOC establishment and full functionality[4, 5].

The PHEOC framework highlights that a fully functional PHEOC is achieved and maintained by putting in place four key components: policies, plans, and procedures; information system and data standard; skilled human resources; and communication technology and physical infrastructure. Africa has made considerable strides in establishing PHEOCs by putting in place these key components to strengthen emergency management. Findings from regional-level meetings on PHEOC implementation and other exchanges including JEE revealed that there are still gaps in establishing functional PHEOCs in the continent. At present, there is no detailed assessment done on the status of PHEOC establishment and functionality. Therefore, WHO in partnership with Africa CDC, WAHO, US CDC, United Kingdom Health Security Agency (UKHSA), Bill and Melinda Gates Foundation (BMGF), and Robert Koch Institute (RKI) conducted a survey to assess the implementation status of core components required to having functional PHEOCs in Africa. The findings of this survey could serve as a resource for planning considerations, generate evidence to contextualize minimum and core requirements for functional PHEOCs, and contribute to policy recommendations.

For peer review only - http://bmjopen.bmj.com/site/about/guidelines.xhtml

METHODS

Data collection

A standardized self-assessment tool was developed based on Annex 9 of the PHEOC framework to determine the PHEOC minimum requirements. The tool encompasses 31 indicators (**Table 1**) across the PHEOC core components to assess the implementation status and existing capacities. The response to each variable allowed for a yes or no answer and a comment section to provide additional information if any. In May 2021, the survey tool was shared with MS and was administered via an online KoBo Toolbox and MS completed the questionnaire and submitted it online. The online survey questionnaire was shared with 54 MS in Africa (47 of the 54 are MS of the WHO African Region). Data were collected between May – November 2021.

The main question to be answered by this survey was 'How are the PHEOCs performing in meeting the minimum requirements of the core components for functional PHEOCs in the Africa Region? To allow for a structure, systematic approach, and comparison, different indicators were selected under each core component of PHEOC and included in the survey to assess the operationalization of PHEOCs across the Region.

The survey intended to answer the following questions

- What are the capacities of the PHEOCs in terms of communication technology and physical infrastructure?
- What are the key PHEOC policies, plans, and procedures for emergency management (e.g., legal framework, multi-hazard plan, PHEOC plan/handbook, hazard-specific plans, functional plans, SOPs, etc.) developed and implemented in the PHEOCs?
- What are the capacities of the PHEOCs in terms of the routine and surge emergency management workforce?
- How is data and information applied for decision-making in the PHEOCs?

Table 1. Indicators assessed in the PHEOC survey by PHEOC core component in Africa

Core components of a PHEOC	***# indicators assessed***
PHEOC policies, plans, and procedures	12
Human resource, training, and simulation exercise	7
Information management and data standards	4
Communication technology and physical infrastructure	8
Total	31

Data analysis

The minimum capabilities required in a PHEOC to run the critical emergency response functions were selected from the parameters included in the survey. The selection of parameters was done based on the WHO framework for a PHEOC and expert consultation. As a result, 19 of the 31 (**Table 2**) parameters from the four core components of a PHEOC were prioritized and classified the PHEOCs into three categories for planning purpose. PHEOCs that meet 80% and above the minimum requirements (n>=15 of the 19) labeled as fully functional, PHEOCs that meet 51%-79% (n=9 to 14 of the 19) as functional, and PHEOCs that meet 50% and below (n<=8 of the 19) labeled as partially functional.

Microsoft Excel 2019 was used to analyze the data.

For peer review only - http://bmjopen.bmj.com/site/about/guidelines.xhtml

Table 2. List of 19 parameters used to define the fulfillment level of the minimum capabilities by core components of a PHEOCs in Africa

PHEOC policy, plans, and procedures	Human resource, training, and simulation exercise	Information management and data standards	Communication technology and physical infrastructure
The legal framework authorizes a PHEOC at the national level	PHEOC has the minimum requirements for routine staff (PHEOC manager and key IMS)	A direct link to the national surveillance structure exists	Dedicated PHEOC facility with adequate workstations for the key IMS functions
Legal frameworks include governance structure, core functions, & authority	Staff trained in PHE preparedness and response	Essential data systematically flow to the PHEOC from relevant sectors	Availability of internet access for all workstations and meeting rooms
Relationships, before, during, and after a PHE between stakeholders defined	PHEOC has a comprehensive exercise program involving more than one per year	Able to collect and manage operational information to inform leadership	Availability of electricity with backup power
A policy group to provide strategic guidance established	Staff are routinely trained on guides, plans, and through simulation to validate competencies	PHEOC uses digital solutions to process its information	
Handbook for PHEOC operations and management is in place	Could contact a roster of trained personnel to fill IMS positions when PHEOC activated		
The multi-hazard response plan is approved			
A business continuity plan (BCP) is in place			

Patient and Public involvement

None.

RESULTS

A total of 51 (93%) MS in the African continent responded to the survey. Forty-one (80%) MS responded that they have established a PHEOC at the national level and 21 MS had a PHEOC at the sub-national level. Within these, 41 of the 47 MS in the WHO African Region responded and 36 MS had a national PHEOC, whereas 17 MS were at the sub-national level.

Table 3 demonstrates that 12 (29%) of the 41 PHEOCs in the continent and 11 (31%) PHEOCs in the AFRO Region met 80% or more of the minimum requirements for the four core components of a PHEOC and labelled as fully functional. In addition, twenty (49%) of the PHEOCs in the continent met 51%-79% and 9 (16%) met 50% and below the minimum requirements and labeled as functional and partially functional, respectively. The number of PHEOCs in the continent that meet the minimum requirements by core components of a PHEOC are displayed in **Table 3**.

For peer review only - http://bmjopen.bmj.com/site/about/guidelines.xhtml

Table 3. Public health emergency operations center minimum requirements fulfillment by core component in Africa, 2021

Core components of a PHEOC	*Fully functional: PHEOCs that met 80% and above (`>=15 of the 19 minimum requirements) # (%)*	*Functional: PHEOCs that met 51%-79% (10 to 14 of the 19 minimum requirements) # (%)*	*Partially functional: PHEOCs that met 50% and Below (<=9 of the 19 minimum requirements) # (%)*
PHEOC policies, plans, and procedures	10 (24)	17 (41)	14 (34)
Human resource, training, and simulation exercise	11 (27)	12 (29)	17 (41)
Information management and data standards	24 (59)	10 (24)	7 (17)
Communication technology and physical infrastructure	18 (44)	11 (27)	12 (29)
Total (n=41)	**12 (29)**	**20 (49)**	**9 (22)**

Core component 1: PHEOC policies, plans, and procedures

Twenty-five (61%) of the PHEOCs have already developed and enacted a legal framework to establish a PHEOC and 23 (56%) of the PHEOC legal frameworks have covered governance structure, core functions, and the scope of authority (**Figure 1**). In addition, twenty-nine (71%) of the PHEOCs had a policy group to provide strategic guidance for the PHEOC operationalization.

Twenty (49%) of the PHEOCs implemented a handbook for PHEOC operations and management to guide their day-to-day operations. Twenty-five (61%) have developed a national multi-hazard response plan that includes the concept of operations addressing priority risks and 19 (46%) of them were endorsed. In 24 (59%) of the PHEOCs, a response plan covering various stakeholders' roles and responsibilities supporting response efforts was in place. Thirty-one (76%) of the PHEOCs had IMS or comparable response structure and 25 (61%) of the PHEOCs could activate within 120 minutes as per JEE recommendations (**Table 4**).

In the WHO African Region, 18 (44%) MS had a handbook for PHEOC operations and management. Besides, 17 (41%) and 23(56%) MS had an approved multi-hazard response plan and the capacity to activate within 120 minutes, respectively.

Table 4. PHEOCs with the capacity for PHEOC plans and procedures, core component 1 (PHEOC policies, plans, and procedures) in Africa, 2021

PHEOC plans and procedures	*Number (#)*	*Of the PHEOCs (%)*
Operational IMS or comparable structure in place	31	76
Multi-hazard response plan including the concept of operations that have been developed	25	61
Could activate the PHEOC within 120 minutes after the decision made	25	61
A response plan that covers the roles and responsibilities of orgs. supporting the response is in place	24	59
Business Continuity Plan (BCP) is in place	23	56

For peer review only - http://bmjopen.bmj.com/site/about/guidelines.xhtml

PHEOCs that implemented handbook for operations and management	20	49
Multi-hazard response plan including the concept of operations that have been approved	19	46

Core component 2: Human resource, training, and simulation exercise

Thirty-eight (93%) of the PHEOCs indicated that at least one manager or focal point was present to oversee day-to-day operations and management of the PHEOCs and 26 (63%) had the minimum expected personnel: PHEOC manager, lead for operations, planning, logistics, finance and administration, communications officer, and ICT expert) to carry out routine preparedness activities. In 17 (41%) PHEOCs, staff were frequently oriented on PHEOC guides, and emergency management, and 14 (34%) PHEOCs regularly simulated exercises (SimEx) to test different capacities. When the IMS was activated, 32 (78%) PHEOCs were able to contact a roster of pre-trained experts to assist with response coordination activities (**Table 5**).

In the WHO African Region, 21 (51%) PHEOCs had the minimum expected routine staff, and 29 (71%) PHEOCs had staff trained on PHE preparedness and response.

Table 5. PHEOCs with the capacity for core component 2 (human resources and simulation exercises) in Africa, 2021

Parameters under human resource, training, and simulation exercise	***Number (#)***	***Of the PHEOCs (%)***
PHEOCs with a designated PHEOC Manager	38	93
Trained staff on PHE preparedness and response	33	80
PHEOCs could contact a roster of trained personnel	32	78
PHEOCs with personnel to run response functions 24/7	26	63
*PHEOCs with the minimum routine staff for IMS functions**	26	63
PHEOCs with a dedicated training program	21	51
Routinely trained staff on existing PHEOC guidance documents and conducted exercises to validate competencies	17	41
PHEOCs with more than one exercise program per year and produces after-action reports	14	34

NB. *PHEOC manager and leads for operations, planning, logistics, finance and administration, communications officer, & ICT manager.

Core component 3: Information management and data standards

There was a digital solution to process the data and information acquired from various sources in 33 (80%) of the PHEOCs. Data were routinely coming from relevant sectors (e.g., surveillance and/or local health systems) in 32 (78%) PHEOCs. Thirty-five (85%) PHEOCs could collect and manage operational information (situational awareness) to inform action whereas 26 (63%) PHEOCs were producing visual dashboards to convey a concise picture of the response and situation.

Core component 4: Communication technology and physical infrastructure

As indicated in **Figure 2**, 27 (66%) of the PHEOCs had sufficient computer workstations, whereas 33 (80%) had workstations capable of serving the essential IMS personnel. Twenty-six (63%) had internet connectivity for the workstations and meeting rooms and 27 (66%) PHEOCs could hold a teleconference.

Twenty-eight (68%) and 21 (51%) PHEOCs with space for at least the IMS staff and adequate internet access, respectively, were from MS of the WHO African Region.

For peer review only - http://bmjopen.bmj.com/site/about/guidelines.xhtml

DISCUSSION

The authors present findings on implementation status of PHEOCs at national levels in Africa from the survey between May to November 2021 and was driven by the aim of assessing the progress made in establishing a PHEOC and implementation status of the core components that make a PHEOC functional. Besides, it was driven by the interest to have resource document to facilitate evidence-based planning and support towards having functional PHEOCs in all MS in the continent. The findings showed that majority of the countries in the continent have designated a PHEOC either in a temporary or permanent facility. The findings revealed that countries made efforts in implementing the four core components of a PHEOC including developing plans and procedures, train experts on PHEOC operations and IMS, strengthening information management and equipping a PHEOC with ICT infrastructure. However, the implementation level of the core components varies from MS to MS.

According to the findings, MS made significant progress and 41 (80%) of them established a PHEOC since the commencement of the regional initiative in 2015 with the aim to support MS to establish PHEOCs to strengthen their emergency management capacity. This initiative was further intensified following the recommendations from 2014-2016 EVD outbreak in West Africa and different stakeholders joined the effort to strengthen emergency preparedness and response capabilities of MS[6]. The results, however, showed that only 12 (29%) of the PHEOCs in the Region met at least 80% of the minimum requirements and are fully functional. Furthermore, 20 (49%) and 9 (16%) of the PHEOCs in the continent met 51%-79% and 50% and below the minimum requirements and labeled as functional and partially functional, respectively. The capacity related to the basic indicators, however, differed. For example, in most of the MS, the core components of PHEOC policies, plans, and procedures and the PHEOC workforce appear to have a critical gap, where only 10 (24%) and 11 (27%) met 80% and above of the minimum requirements. The main reason for this could be technical capacity, financial or other constraints. Most of the MS, on the other hand, had the required capability in terms of core component 3, Information management, and data standards.

Adverse health outcomes and economic disruption attributed to PHEs requires effective preparedness and response procedures[2,10]. A legal framework for approving the PHEOC's establishment and operation, a handbook for its operations and management, event or hazard-specific response and management plans, an incident action plan, and other pertinent plans and procedures must ideally be in place before any PHE occurs[7]. Among the key indicators under the core component of PHEOC policies, plans, and procedures, only 20 PHEOCs had a handbook for PHEOC operations and management and a multi-hazard response plan including the concept of operations. Besides, a business continuity plan (BCP) was in place in only 23 PHEOCs. This might affect the effectiveness of the PHEOC to coordinate response during emergencies due to the absence of clear guidance and operational response plans.

Regarding the workforce capacity, despite most of the PHEOCs met the key requirements for some requirements, there were gaps in having a dedicated training program to train routine PHEOC and surge staff. Only 41% PHEOCs had routinely trained staff on existing PHEOC guidance documents and conducted exercises to validate competencies. Besides, 34% of the PHEOCs had more than one exercise program per year and prepared after-action reports to address the gaps identified from the exercise. Though the personnel needed for a PHEOC vary by many factors, including the emergency's size and complexity, a PHEOC usually has a routine staff responsible for daily activities while a surge personnel will be mobilized when the PHEOC is activated. The surge personnel, therefore, should be identified and trained in IMS and specific functions prior to PHE occurs and an up-to-date roster should be maintained[11]. When the PHEOC is activated, it should be staffed with a team of subject matter experts drawn from the roster and a regular training and exercises should be conducted to determine the function, staffing, and ensure availability of a trained and skilled personnel [11-15].

An EOC's lifeblood is information. Information management is a critical component of a functional PHEOC, and it entails gathering, analyzing, and distributing data promptly[8-12, 16]. Event-specific data, event management information, and context data are the three categories of data that must be

For peer review only - http://bmjopen.bmj.com/site/about/guidelines.xhtml

consistently recorded, analyzed, and displayed in a PHEOC[7]. During response coordination, PHEOC requires specific types of data depending on the type of PHE. Data should be gathered according to local conditions. During activation, EOCs use data technologies and informal networks of public health professionals to monitor epidemiologic data and field reports from several sources[13]. In addition, the PHEOC should maintain clear and updated information about the incident or disaster. Effective communication is mandatory to keep the public trust in the messages and the function of the PHEOC[17].

Majority of the PHEOCs had better performances for most indicators under the core component of communication technology and physical infrastructure. However, there were critical gaps in having interoperable radio communication and logistic facilities. The primary reason for the gaps observed might be limitations in internet accessibility, funding, and technical capabilities. This primarily affected the effectiveness of the PHEOC functionality in terms of ease communication, especially at the sub-national level. Furthermore, the findings demonstrated that most of the MS have a PHEOC capacity only at the national level and there are still gaps at the sub-national level where most emergencies are managed. Therefore, addressing such gaps might be critically considered in MS with lower capacity. The PHEOC should have the potential to acquire the technologies such as computers, phones, TV plasma screens, projectors, and radio systems that support telecommunications, information management, and visualization of operational information resulting in more effective response coordination[18].

LIMITATION

The survey was self-administered hence respondents were likely not to adjudge performance status on the same scale. The largely binary nature of the questionnaire (yes/no options) may have limited the documentation of minor but important progress made.

CONCLUSION

Member States made significant progress in setting up a PHEOC to improve the emergency management capabilities and 41 (80%) MS established a PHEOC since the commencement of the regional initiative in 2015. Of these, one-fifth (12) MS have a national PHEOC that met 80% or more of the minimum requirements to operate critical emergency functions and labelled as fully functional. In addition, 20 (49%) and 9 (16%) MS have PHEOCs that met 51%-79% and 50% and below the minimum requirements and categorized as functional but need improvement and partially functional respectively. However, the PHEOCs in many MS are of different capabilities and need improvement to meet the requirements to be fully functional and some MS still have no PHEOCs.

The main bottlenecks for implementing functional PHEOCs meeting the requirements in all the four core components in Africa include the absence of a legal framework that clearly defines its mandate and functions, the lack of a standing policy group to provide operational support and strategic direction, unapproved plans and procedures, and limited skilled human resources and funding for operations and sustainability.

Therefore, it is crucial to enhance the implementation of functional PHEOC according to the WHO framework and accompanying handbooks and guides. The implementation planning processes require the involvement of all relevant stakeholders. Besides, regional, and international partners need to support MS' efforts to address these gaps in developing functional PHEOC.

Contributors

ST, WM, WE, VK, YK, AA, JL, MT, CM, ER, VB, EC, LM, AH, MA, IC, FB, and AG prepared the survey tool. ST and AL conducted relevant article searches, developed a data analysis plan, analyzed the data, and wrote the manuscript. ST, AL, WM, WE, VK, YK, AA, JL, MT, CM, ER, VB, EC, LM, AH, MA, IC, FB, and AG contributed to the writing and reviewed the final manuscript. All authors read and approved the final manuscript.

Funding statement

This work received no specific grant from any funding agency in the public, commercial or not-for-profit sectors.

For peer review only - http://bmjopen.bmj.com/site/about/guidelines.xhtml

Competing interests

None declared.

Ethics Statement

Patients were not involved, and ethical approval was not required.

Availability of data and materials

The datasets from the survey used in this article are available upon reasonable request.

Acknowledgment

We thank all the MS in the continent for their significant contributions to the survey. We also want to thank everyone who helped review the survey tool and article.

REFERENCES

1. Health S, Centre O. Afro shoc 1. 2014. 1–67 p.
2. Talisuna AO, Okiro EA, Yahaya AA, Stephen M, Bonkoungou B, Musa EO, et al. Spatial and temporal distribution of infectious disease epidemics, disasters, and other potential public health emergencies in the World Health Organisation Africa region, 2016-2018. Global Health. 2020;16(1):1–13.
3. WHO. This booklet introduces all 11 sections of the Integrated Disease Surveillance and Response (IDSR) Technical Guidelines. 2019;
4. Kamradt-Scott A. The International Health Regulations (2005). Int Organ Law Rev. 2019;16(2):242–71.
5. WHO. Public health events of initially unknown etiology : A framework for preparedness and response in the African Region. 2014; Available from: http;//www.who.int/iris/handle/10665/112832
6. Agbo S, Gbaguidi L, Biliyar C, Sylla S, Fahnbulleh M, Dogba J, et al. Establishing National Multisectoral Coordination and collaboration mechanisms to prevent, detect, and respond to public health threats in Guinea, Liberia, and Sierra Leone 2016–2018. One Heal Outlook. 2019;1(1):1–13.
7. WHO. Framework for a Public Health Emergency Operations Centre. MMWR Morb Mortal Wkly Rep [Internet]. 2015;50(RR13)(July 27):1–35. Available from: www.who.int/about/licensing/%0Ahttps://apps.who.int/iris/bitstream/handle/10665/196135/9789241565134_eng.pdf?sequence=1
8. WHO; A Systematic Review of Public Health Emergency Operations Centres (EOC). 2013;(December):11.
9. Network of Public Health Emergency Operation Centers established to protect lives _ WHO _ Regional Office for Africa.
10. World Health Organization. Strengthening health-system emergency preparedness. 2012;96. Available from: http://www.euro.who.int/__data/assets/pdf_file/0008/157886/e96187.pdf
11. Xu M, Li SX. Analysis of good practice of public health Emergency Operations Centers. Asian Pac J Trop Med [Internet]. 2015;8(8):677–82. Available from: http://dx.doi.org/10.1016/j.apjtm.2015.07.006
12. FEMA. National Incident Management System 2017 learning materials. 2017;
13. Balajee SA, Pasi OG, Etoundi AGM, Rzeszotarski P, Do TT, Hennessee I, et al. Sustainable model for public health emergency operations centers for global settings. Emerg Infect Dis. 2017;23(December): S190–5.
14. CDC. CDC Emergency Operations Center | CDC. CDC Emergency Operations Center | CDC. 2020.
15. Olu O, Usman A, Kalambay K, Anyangwe S, Voyi K, Orach CG, et al. What should the African health workforce know about disasters? Proposed competencies for strengthening public health disaster risk management education in Africa. BMC Med Educ. 2018;18(1).
16. Scanlon J. The Role of EOCs in Emergency Management: A Comparison of American and Canadian Experience. Vol. 12, International Journal of Mass Emergencies and Disasters. 1994.

For peer review only

For peer review only - http://bmjopen.bmj.com/site/about/guidelines.xhtml

p. 51–75.
17. Rebmann T, Carrico R, English JF. Lessons public health professionals learned from past disasters. Public Health Nurs. 2008;25(4):344–52.
18. Simon R, Teperman S. WTC Lessons for disaster management. 2001;5(6):5–7.

For peer review only

For peer review only - http://bmjopen.bmj.com/site/about/guidelines.xhtml

List of Figures

Figure 1. PHEOCs with the capacity for PHEOC legal frameworks, core component 1 (PHEOC policies, plans, and procedures) in Africa, 2021

Figure 2. PHEOCs with the capacity for core component 4 (communication technology and physical infrastructure) minimum requirements in Africa, 2021

For peer review only

Figure 1. PHEOCs with the capacity for PHEOC legal frameworks, core component 1 (PHEOC policies, plans, and procedures) in Africa, 2021

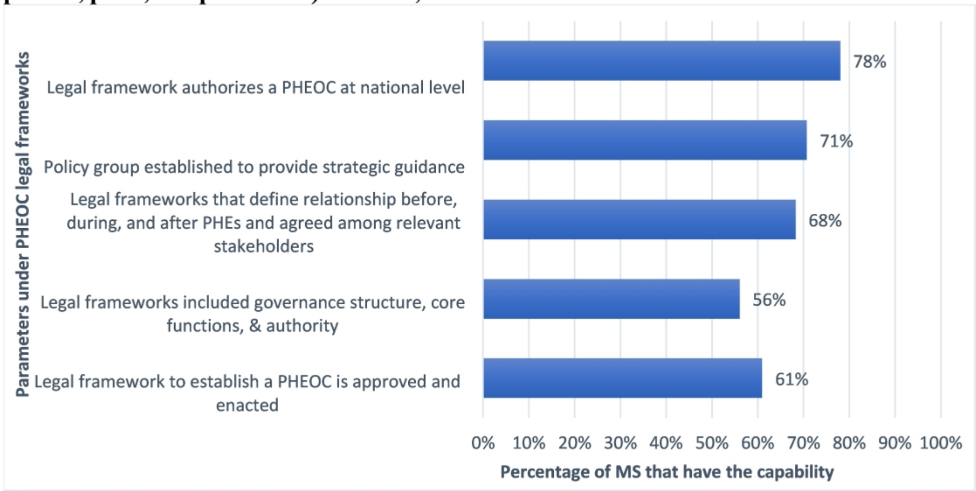

165x92mm (600 x 600 DPI)

Figure 2. PHEOCs with the capacity for core component 4 (communication technology and physical infrastructure) minimum requirements in Africa, 2021

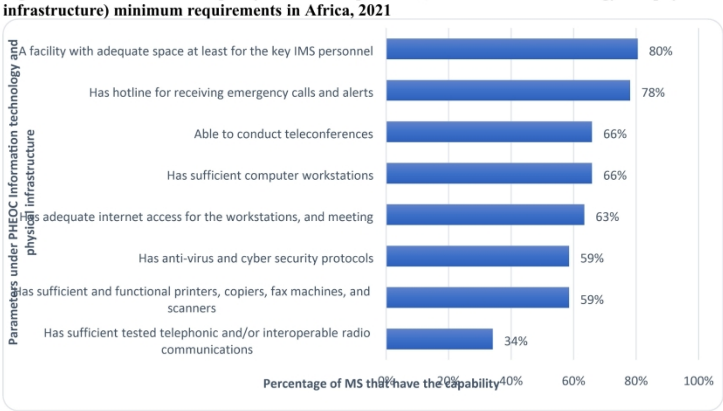

215x279mm (300 x 300 DPI)

ANNEX 9: Checklist for Implementing a PHEOC

In this checklist, *'capabilities'* means that all the material and human resources with the required knowledge, skills and abilities are available with the necessary policy authority and procedural instructions.

	PHEOC PLANNING GUIDANCE	
	**Legal authority**	**Comments**
1	☐ Legal authority for PHEOC established<br>☐ Government commitment has been secured<br>☐ Public health emergencies are recognised as potential national disasters	
2	☐ The collaboration mechanism between the Ministry of Health and the National Disaster Management Organization, other Ministries, agencies and sectors during public health emergencies has been defined and agreed	
3	☐ Mandate and scope of PHEOC decisions and operations approved by government, partners and stakeholders	
	**Policy group**	
4	☐ A policy group to provide policy guidance to PHEOC has been established	
	**Steering committee**	
5	☐ A steering committee of PHEOC stakeholders has been established for the planning and development of PHEOC	
6	☐ Hazards, vulnerabilities and resulting risks have been identified and prioritized	
7	☐ PHEOC objectives have been developed	
	**Planning**	
8	☐ An all-hazards national public health emergency management plan, addressing priority risks, has been developed and approved	
9	☐ Emergency Response Plan for the health sector is in place.	
10	☐ Response plans detail roles and responsibilities for MoH and other response agencies, sectors and jurisdiction at various levels in the response organization, including private sector and NGO organizations.	
11	☐ Response plans describe scaled levels of response with resource requirements for each level and procedures for acquiring additional resources	
12	☐ Response plans detail the notification, reporting, engagement and coordination requirements	
13	Response plans contain information about laboratories including:<br>☐ Contact information<br>☐ Types, e.g. biosecurity level, locations, business hours, contact information and links to MoH surveillance systems<br>☐ Types of specimens or samples tested<br>☐ Types of testing provided<br>☐ Standard Operating Procedures for collection, packaging, shipping and maintaining chain of custody of specimens and samples.	

14	Response Plans contain verified location, contact and emergency response information for:<br>☐ Hospitals, clinics and treatment centres<br>☐ Points of entry<br>☐ Pharmacies<br>☐ NGO's in-country<br>☐ Public health units<br>☐ Social services offices<br>☐ Shelters<br>☐ Partner Government agencies<br>☐ IHR Focal Point and WHO IHR contact point<br>☐ Other subnational PHEOCs or National coordination centre	
15	☐ Response plans provide SOPs for coordinating with law enforcement and national security agencies.	
	**IMPLEMENTING A PHEOC**	
16	☐ A clear operational structure based on the IMS and comprising (i) Management, (ii) Operations, (iii) Planning, (iv) Logistics and (v) Finance and Administration functions is in place<br>☐ Staff, trained in emergency response management and mission-critical public health activities and objectives, are available to fill key PHEOC roles at all times<br>☐ A roster of incident managers is maintained<br>☐ The PHEOC facility manager has been appointed<br>☐ The information manager has been appointed	
17	The PHEOC has the capability to:<br>☐ prepare public health alerts<br>☐ conduct web surveillance to detect and correct of rumours, public and interest group concerns and media misinformation	
18	☐ Infrastructure, personnel and procedures are in place sufficient to support IHR (2005) notification requirements (surveillance, detection, reporting, IHR focal point)<br>☐ The PHEOC has the capability to produce and share a common operational picture	
19	☐ The PHEOC has the capability to direct and support logistical operations for the acquisition, storage, transportation and delivery of PPE, medical equipment, pharmaceuticals, laboratory supplies and medical countermeasure supplies for public health emergency responses in country	
20	☐ The PHEOC has the capability to provide logistical and operational support for team(s) and protect the health and safety of deployed teams	
21	☐ Administrative policy has been formulated to support emergency contracting, hiring, procurement and management of donor funds.	

For peer review only - http://bmjopen.bmj.com/site/about/guidelines.xhtml

	CORE COMPONENTS OF A PHEOC	
	**Plans and procedures**	
22	☐ PHEOC plans (EOC plan and CONOPS) have been approved	
23	The PHEOC plan/handbook for staff includes:<br>☐ A concept of operations<br>☐ Map of the PHEOC workstations, rooms and inventories of equipment<br>☐ Routine staffing requirements<br>☐ Standard operating procedures<br>☐ Forms and templates for data collection, reporting, briefing etc<br>☐ Documentation and records management processes<br>☐ Role descriptions and job aids for PHEOC functional positions<br>☐ Response levels and thresholds<br>☐ Activation, scaling, deactivation thresholds and procedures<br>☐ Contact information for key officials and PHEOC personnel<br>☐ Notification and communication protocols with host agency, response organizations and partner agencies.	
24	☐ Procedures and equipment are in place to establish and maintain communications between the IHR Focal Point and WHO Regional and Headquarters offices	
25	☐ Where relevant, hazard-specific response annexes have been developed to address chemical, infectious disease, radiological or food and water safety threats.	
26	☐ Procedures in place for credentialing and permitting access for health professionals from other jurisdictions to operate in-country	
27	The PHEOC has a Business Continuity Plan (Continuity of Operations Plan) which includes:<br>☐ Priority functions that need to be maintained<br>☐ Key personnel that are needed to implement the plan<br>☐ Alternative/backup PHEOC site(s) and relocation plans<br>☐ Records and data management procedures<br>☐ Processes for maintaining critical external communications<br>☐ Activation, notification and deactivation procedures	
28	The PHEOC has a communications plan for emergency public information and warning that outlines:<br>☐ Triggers for issuing information to general public or specific audiences<br>☐ Message templates for priority threats<br>☐ Roles and responsibilities for communications staff<br>☐ Procedures for developing and approving new messages<br>☐ Procedures for issuing risk communications using traditional media, official social media accounts and agency website posting.<br>☐ Process for developing linguistically and culturally appropriate messages.<br>☐ Approval authorities for external messages.	

For peer review only

29	☐ Deactivation and demobilization plans describe procedures for notification, closing procedures, debriefings, records management, restoring and repatriating staff and supplies to pre-incident levels or to regular roles.	
	**Physical infrastructure**	
30	☐ A PHEOC suitable facility is available<br>☐ A multi-use facility can be converted in one hour to an adequate operational PHEOC<br>☐ A suitable facility has been acquired but not yet developed as an operational PHEOC	
31	☐ The PHEOC meets requirements for accessibility, security, structural integrity and resistance to natural and human generated hazards.<br>☐ The PHEOC has adequate space for the all expected PHEOC functions, private meetings, surge staff, secure communications, IT equipment and support personnel<br>☐ The PHEOC has audiovisual functionality to project operational, contextual and event status information, tested and maintained	
32	The PHEOC has:<br>☐ sufficient potable water supply and adequate water to address sanitary requirements<br>☐ structural maintenance, janitorial and waste removal services<br>☐ toilet and sanitary facilities scaled for the expected occupancy<br>☐ approved quantity of first aid supplies<br>☐ an approved fire suppression system and/or equipment<br>☐ a staff evacuation plan<br>☐ security measures to control access<br>☐ a backup site that can be activated if the primary site becomes untenable	
	**Information and communications technology (ICT) infrastructure**	
33	☐ The PHEOC has sufficient computer workstations with necessary application software loaded and tested.	
34	☐ PHEOC servers and backups, with needed applications are maintained and routinely tested	
35	☐ The PHEOC has sufficient tested telephonic and/ or interoperable radio communications for every workstation and meeting space, with spares.	
36	☐ There is tested web or video conferencing equipment in a private meeting space.	
37	☐ There are sufficient printers, copiers, fax machines and scanners are maintained and functional.	
38	☐ There is sufficient quantity of electricity including backup capacity (generator and fuel, UPS for critical data storage and processing)	
39	☐ The facility has an HVAC system sufficient to maintain comfort for occupants and keep IT equipment cool.	

For peer review only - http://bmjopen.bmj.com/site/about/guidelines.xhtml

	**Information systems and data standards**	
40	The PHEOC has the capability to receive, analyse, display, report and share reports of reportable and unusual diseases and health conditions from:<br>☐ public and private sector healthcare providers and facilities<br>☐ sub-national offices and units<br>☐ veterinary and animal health sources<br>☐ points of entry<br>☐ NGOs<br>☐ Other national governments and international agencies<br>☐ Other arms and branches of government and Community based sources	
41	☐ The PHEOC has the capability to collect, process and share field epidemiological and other investigational data (including (i) receiving, aggregating and analyzing field data, and (ii) visualization of epidemiological information and timely preparation of reports in standardized format for decision making and sharing with partners)	
42	☐ The PHEOC is linked to a national surveillance information structure for monitoring and responding to priority risks.	
43	The PHEOC has the capability to:<br>☐ receive and share public health laboratory data related to outbreaks and events<br>☐ receive, produce and share integrated surveillance information containing epidemiological findings and laboratory results at individual and aggregated levels<br>☐ provide data analytic support for other events of public health interest (eg: mass gatherings)<br>☐ produce geospatial information such as maps and other visualizations from common operational datasets<br>☐ ascertain the status and report key external partner/resource information such as hospital bed availability, treatment centres, laboratories etc.<br>☐ monitor the status and needs of deployed field teams and other responder personnel including assisting international support and NGOs<br>☐ display contextual operational information such as population distribution, administrative and political boundaries, transportation infrastructure, hydrology and elevations	
44	☐ The PHEOC datasets include identification of information necessary to develop a common operational picture for priority risks	
45	☐ The PHEOC can access, display and track status information regarding affected persons and vulnerable populations	
46	☐ The PHEOC has the capability to monitor and account for all resources utilized in a response	
47	☐ The PHEOC has the capability to track and display the status of tasks and objectives	
48	☐ Staff filling IMS functional roles are trained to use relevant software	

For peer review only

	**Human Resources**	
49	☐ The PHEOC has designated non-emergency staffing to manage the facility	
50	☐ Technical personnel are available for the operation, maintenance and repair of audio-visual, telecommunications and computer equipment.	
51	☐ There are personnel trained in emergency management and PHEOC standard operating procedures	
52	☐ There is sufficient trained staff to activate the PHEOC on short notice	
53	☐ The PHEOC has the capability to identify and contact a roster of trained personnel, equipment and supplies for deployment to field sites responders (e.g. rapid response teams)	
	**TRAINING AND EXERCISES**	
54	☐ The PHEOC has a dedicated training program based on a training needs assessment for incident management personnel that addresses staff roles during response operations; utilization of communications and data processing equipment and software; and hazard-specific response knowledge.	
55	☐ The PHEOC has a comprehensive, progressive exercise program for all staff and partners, national and NGO agencies and produces evaluation reports that identify corrective actions required.	
	**MONITORING AND EVALUATION**	
56	☐ The PHEOC training and exercise programs are primary components of a performance monitoring and evaluation system focused on continuous improvement of public health emergency management capability and effectiveness.	
	**COSTING, FUNDING AND SUSTAINING A PHEOC**	
57	☐ The PHEOC plan includes an itemised schedule of costs	
58	☐ There is funding plan and funding mechanism to support the PHEOC	
59	☐ Funds are available to develop and sustain the PHEOC	

For peer review only - http://bmjopen.bmj.com/site/about/guidelines.xhtml

Framework for a Public Health Emergency Operations Centre

November 2015

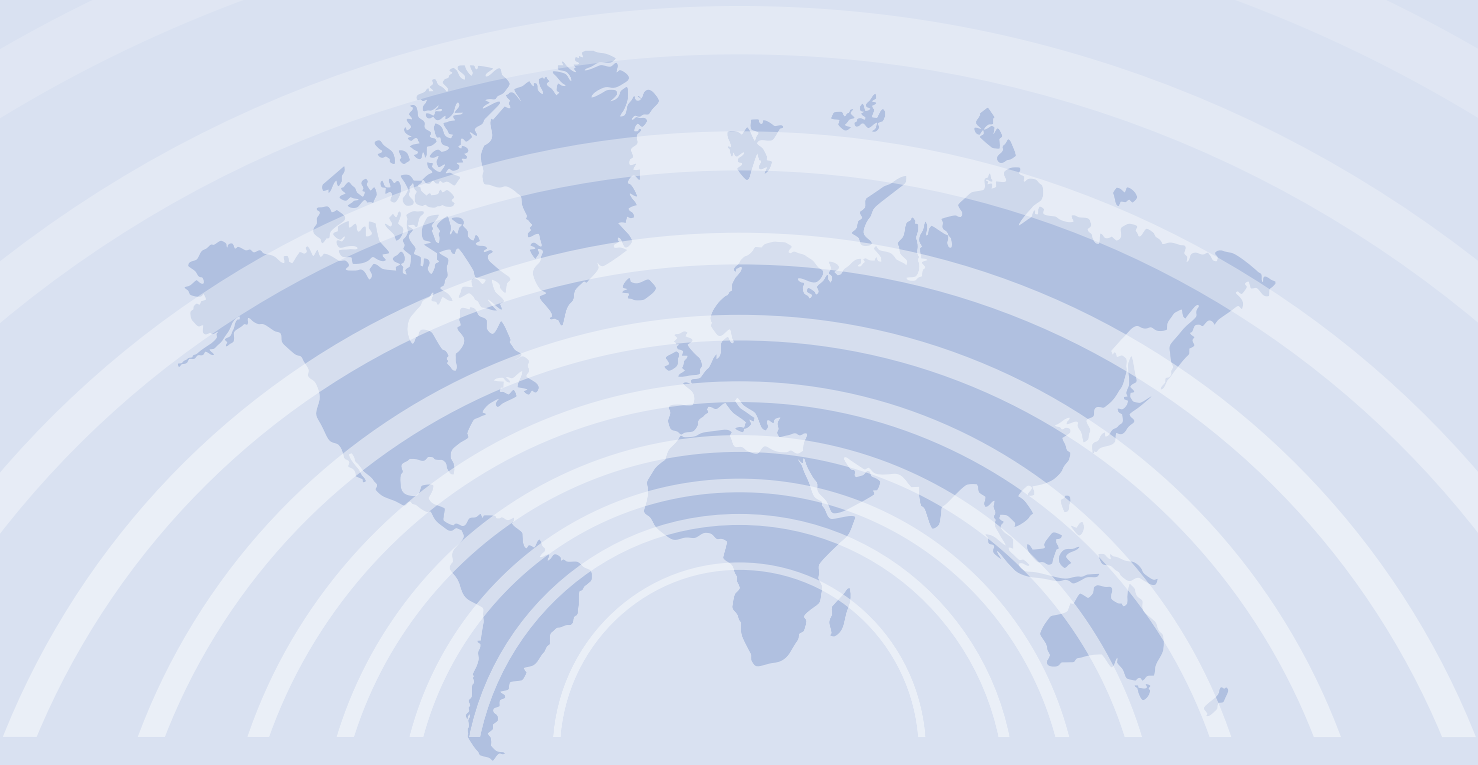

World Health Organization

Framework for a Public Health Emergency Operations Centre

November 2015

World Health Organization

For peer review only

WHO Library Cataloguing-in-Publication Data

Framework for a Public Health Emergency Operations Centre. November 2015

I.World Health Organization.

ISBN 978 92 4 156513 4

Subject headings are available from WHO institutional repository

For peer review only

© World Health Organization 2015

All rights reserved. Publications of the World Health Organization are available on the WHO web site (www.who.int) or can be purchased from WHO Press, World Health Organization, 20 Avenue Appia, 1211 Geneva 27, Switzerland (tel.: +41 22 791 3264; fax: +41 22 791 4857; e-mail: bookorders@who.int).

Requests for permission to reproduce or translate WHO publications –whether for sale or for non-commercial distribution– should be addressed to WHO Press through the WHO website (www.who.int/about/licensing/copyright_form/en/index.html).

The designations employed and the presentation of the material in this publication do not imply the expression of any opinion whatsoever on the part of the World Health Organization concerning the legal status of any country, territory, city or area or of its authorities, or concerning the delimitation of its frontiers or boundaries. Dotted lines on maps represent approximate border lines for which there may not yet be full agreement.

The mention of specific companies or of certain manufacturers' products does not imply that they are endorsed or recommended by the World Health Organization in preference to others of a similar nature that are not mentioned. Errors and omissions excepted, the names of proprietary products are distinguished by initial capital letters.

All reasonable precautions have been taken by the World Health Organization to verify the information contained in this publication. However, the published material is being distributed without warranty of any kind, either expressed or implied. The responsibility for the interpretation and use of the material lies with the reader. In no event shall the World Health Organization be liable for damages arising from its use.

Printed in Switzerland

Design and layout: Jean-Claude Fattier

STROBE Statement—Checklist of items that should be included in reports of ***cross-sectional studies***

	Item No	Recommendation	
**Title and abstract**	1	(*a*) Indicate the study's design with a commonly used term in the title or the abstract	Page 1, line 1-3
		(*b*) Provide in the abstract an informative and balanced summary of what was done and what was found	Page 2, line 1-26
**Introduction**			
Background/rationale	2	Explain the scientific background and rationale for the investigation being reported	Page 3, 1-44
Objectives	3	State specific objectives, including any prespecified hypotheses	Page 3, line 45-50
**Methods**			
Study design	4	Present key elements of study design early in the paper	Page 4, line 1-5
Setting	5	Describe the setting, locations, and relevant dates, including periods of recruitment, exposure, follow-up, and data collection	Page 4, line 6-25
Participants	6	(*a*) Give the eligibility criteria, and the sources and methods of selection of participants	Page 4, 1-5
Variables	7	Clearly define all outcomes, exposures, predictors, potential confounders, and effect modifiers. Give diagnostic criteria, if applicable	Not applicable
Data sources/ measurement	8*	For each variable of interest, give sources of data and details of methods of assessment (measurement). Describe comparability of assessment methods if there is more than one group	*Not applicable*
Bias	9	Describe any efforts to address potential sources of bias	Not applicable
Study size	10	Explain how the study size was arrived at	Not applicable, no need to determine sample size
Quantitative variables	11	Explain how quantitative variables were handled in the analyses. If applicable, describe which groupings were chosen and why	Not applicable, only quantitative variables taken
Statistical methods	12	(*a*) Describe all statistical methods, including those used to control for confounding	Not applicable.
		(*b*) Describe any methods used to examine subgroups and interactions	Not applicable.
		(*c*) Explain how missing data were addressed	There were no as such missing data
		(*d*) If applicable, describe analytical methods taking account of sampling strategy	Descriptive analysis was done
		(*e*) Describe any sensitivity analyses	
**Results**			
Participants	13*	(a) Report numbers of individuals at each stage of study—eg numbers potentially eligible, examined for eligibility, confirmed	Page 5, line 3-7

For peer review only - http://bmjopen.bmj.com/site/about/guidelines.xhtml

		eligible, included in the study, completing follow-up, and analysed	
		(b) Give reasons for non-participation at each stage	Not applicable
		(c) Consider use of a flow diagram	Not applicable
Descriptive data	14*	(a) Give characteristics of study participants (eg demographic, clinical, social) and information on exposures and potential confounders	Pages 6-7
		(b) Indicate number of participants with missing data for each variable of interest	Not applicable
Outcome data	15*	Report numbers of outcome events or summary measures	Not applicable
Main results	16	(*a*) Give unadjusted estimates and, if applicable, confounder-adjusted estimates and their precision (eg, 95% confidence interval). Make clear which confounders were adjusted for and why they were included	Not applicable
		(*b*) Report category boundaries when continuous variables were categorized	Not applicable
		(*c*) If relevant, consider translating estimates of relative risk into absolute risk for a meaningful time period	Not applicable
Other analyses	17	Report other analyses done—eg analyses of subgroups and interactions, and sensitivity analyses	Not applicable
**Discussion**			
Key results	18	Summarise key results with reference to study objectives	Page 8, line 1-11
Limitations	19	Discuss limitations of the study, taking into account sources of potential bias or imprecision. Discuss both direction and magnitude of any potential bias	Page 9, line 17-20
Interpretation	20	Give a cautious overall interpretation of results considering objectives, limitations, multiplicity of analyses, results from similar studies, and other relevant evidence	This is the first in-depth analysis of PHEOC implementation status, and the study was able to determine the status of implementation of key capacities. The findings could be used for planning considerations.
Generalisability	21	Discuss the generalisability (external validity) of the study results	
**Other information**			
Funding	22	Give the source of funding and the role of the funders for the present study and, if applicable, for the original study on which the present article is based	No specific funding, data collection was done online.

For peer review only

For peer review only - http://bmjopen.bmj.com/site/about/guidelines.xhtml

*Give information separately for exposed and unexposed groups.

Note: An Explanation and Elaboration article discusses each checklist item and gives methodological background and published examples of transparent reporting. The STROBE checklist is best used in conjunction with this article (freely available on the Web sites of PLoS Medicine at http://www.plosmedicine.org/, Annals of Internal Medicine at http://www.annals.org/, and Epidemiology at http://www.epidem.com/). Information on the STROBE Initiative is available at www.strobe-statement.org.

For peer review only

For peer review only - http://bmjopen.bmj.com/site/about/guidelines.xhtml

BMJ Open

Public health emergency operations centres in Africa: a cross-sectional study assessing the implementation status of core components and areas for improvement, December 2021

Journal:	*BMJ Open*
Manuscript ID	bmjopen-2022-068934.R2
Article Type:	Original research
Date Submitted by the Author:	16-Feb-2023
Complete List of Authors:	Fekadu, Senait; World Health Organization Regional Office for Africa<br>Gebrewahid, Abrham Lilay; World Health Organization Regional Office for Africa<br>Mankoula, Wessam; African Union, Division of Emergency Preparedness and Response<br>Eteng, Womi; African Union, Division of Emergency Preparedness and Response<br>Lokossou, Virgil; West African Health Organisation, ECOWAS Regional Center for Surveillance and Disease Control<br>Kawe, Yan; World Health Organization Regional Office for Africa<br>Abdullah, Ali; World Health Organisation Regional Office for the Eastern Mediterranean<br>Jian, L; World Health Organization<br>Tut, Mathew; African Union, Division of Emergency Preparedness and Response<br>Wilton, Menchion Chuck; Centers for Disease Control and Prevention<br>Rosenfeld, Emily; Centers for Disease Control and Prevention<br>Bemo, Valerie; Bill & Melinda Gates Foundation<br>Collard, Emily; UK Health Security Agency<br>McGinley, Liz; UK Health Security Agency<br>Halm, Ariane; Robert Koch Institute<br>Aragaw, Merawi; African Union, Division of Emergency Preparedness and Response<br>Conteh, Ishata Nannie; World Health Organization Regional Office for Africa, Emergency Preparedness and Response<br>Braka , Fiona ; World Health Organization Country Office for Nigeria<br>Gueye, Abdou Salam; World Health Organization Regional Office for Africa, Emergency Preparedness and Response Programme
**Primary Subject Heading**:	Public health
Secondary Subject Heading:	Public health, Health policy
Keywords:	PUBLIC HEALTH, Health policy < HEALTH SERVICES ADMINISTRATION & MANAGEMENT, Information management < BIOTECHNOLOGY & BIOINFORMATICS

SCHOLARONE™
Manuscripts

I, the Submitting Author has the right to grant and does grant on behalf of all authors of the Work (as defined in the below author licence), an exclusive licence and/or a non-exclusive licence for contributions from authors who are: i) UK Crown employees; ii) where BMJ has agreed a CC-BY licence shall apply, and/or iii) in accordance with the terms applicable for US Federal Government officers or employees acting as part of their official duties; on a worldwide, perpetual, irrevocable, royalty-free basis to BMJ Publishing Group Ltd ("BMJ") its licensees and where the relevant Journal is co-owned by BMJ to the co-owners of the Journal, to publish the Work in this journal and any other BMJ products and to exploit all rights, as set out in our licence.

The Submitting Author accepts and understands that any supply made under these terms is made by BMJ to the Submitting Author unless you are acting as an employee on behalf of your employer or a postgraduate student of an affiliated institution which is paying any applicable article publishing charge ("APC") for Open Access articles. Where the Submitting Author wishes to make the Work available on an Open Access basis (and intends to pay the relevant APC), the terms of reuse of such Open Access shall be governed by a Creative Commons licence – details of these licences and which Creative Commons licence will apply to this Work are set out in our licence referred to above.

Other than as permitted in any relevant BMJ Author's Self Archiving Policies, I confirm this Work has not been accepted for publication elsewhere, is not being considered for publication elsewhere and does not duplicate material already published. I confirm all authors consent to publication of this Work and authorise the granting of this licence.

For peer review only

Public health emergency operations centres in Africa: a cross-sectional study assessing the implementation status of core components and areas for improvement, December 2021

[1*]Fekadu Senait Tekeste, [1]Abrham Lilay, [4]Wessam Mankoula, [4]Womi Eteng, [5]Lokossou Virgil, [1]Yan Kawe, [2]Ali Abdullah, [3]Jian Li, [4]Mathew Tut, [5]Chuck Menchion Wilton, [5]Emily Rosenfeld, [8]Valerie Bemo, [6]Emily Collard, [6]Liz McGinley, [9]Ariane Halm, [4]Merawi Aragaw, [1]Conteh Ishata Nannie, [1]Braka Fiona, [1]Gueye Abdou Salam.

Affiliations

[1]World Health Organization Regional Office for Africa, Brazzaville, Republic of Congo
[2]World Health Organization, Regional Office for Eastern Mediterranean, Cairo, Egypt,
[3]World Health Organization, Headquarters, Geneva, Switzerland,
[4]Africa Centres for Disease Control and Prevention, Division of Emergency Preparedness and Response, Addis Ababa, Ethiopia,
[5]ECOWAS, Regional Center for Surveillance and Disease Control, Abuja, Nigeria
[6]US Centers for Disease Control and Prevention, Atlanta, Georgia, USA,
[7]UK Health Security Agency, Emergency Preparedness, Resilience and Response, London, UK,
[8]Bill and Melinda Gates Foundation, Seattle, Washington, USA.
[9]Robert Koch Institute, Crisis Management Unit, Infectious disease epidemiology Department, Berlin, Germany

Email addresses

FST: tekestes@who.int, AL: lilayabrish@yahoo.com, WM: wessamm@africa-union.org, WE: etengw@africa-union.org, VL: vlokossou@wahooas.org, YK: kawey@who.int AA: abdullahal@who.int, JL: ij@who.int, MT: MathewK@africa-union.org, CM: wom0@cdc.gov, EC: emily.collard@ukhsa.gov.uk LM: liz.mcginley@ukhsa.gov.uk, ER: yhc5@cdc.gov, VN: valerie.bemo@gatesfoundation.org, AH: halma@rki.de, MA: merawia@africa-union.org, CIN: contehis@who.int, BF: brakaf@who.int, and GAS: gueyea@who.int.

Corresponding Author

* Fekadu Senait Tekeste, World Health Organization Regional Office for Africa (WHO AFRO), Congo-Brazzaville, email: tekestes@who.int phone no. +242 06 880 5214

For peer review only

ABSTRACT

Objective To assess implementation status of Public Health Emergency Operations Centres (PHEOCs) in all countries in Africa.

Design Cross-sectional.

Setting Fifty-four national PHEOC focal points in Africa responded to an online survey between May and November 2021. Included variables aimed to assess capacities for each of the four PHEOC core components. To assess the PHEOCs' functionality, criteria were defined from among the collected variables by expert consensus based on PHEOC operations' prioritisation. We report results of the descriptive analysis, including frequencies of proportions.

Results A total of 51 (93%) African countries responded to the survey. Among these, 41 (80%) have established a PHEOC. Twelve (29%) of these met 80% or more of the minimum requirements and were classified as fully functional. Twelve (29%) and 17 (41%) PHEOCs that met 60%-79% and below 60% the minimum requirements were classified as functional and partially functional, respectively.

Conclusions Countries in Africa made considerable progress in setting up and improving functioning of PHEOCs. One-third of the responding countries with a PHEOC have one fulfilling at least 80% of the minimum requirements to operate the critical emergency functions. There are still several African countries that either do not have a PHEOC or whose PHEOCs only partially meet these minimal requirements. This calls for significant collaboration across all stakeholders to establish functional PHEOCs in Africa.

Strengths and limitations

- This study collects first-hand information on emergency operations implemented through PHEOCs in African countries during the COVID-19 pandemic.
- The results of this survey illustrate the state of PHEOC implementation in Africa based on an international framework.
- The survey was self-administered hence respondents did possibly not appraise performance status on the same scale.
- The survey does not deliver in-depth information for more detailed insight, as the main purpose was obtaining an overview of the PHEOC implementation status.

For peer review only

For peer review only - http://bmjopen.bmj.com/site/about/guidelines.xhtml

INTRODUCTION

Public health threats, for example stemming from natural disasters, and consequent public health emergencies (PHEs) continue to be a major concern for African countries. In Africa, over 100 PHEs are recorded annually, 80% or more are caused by infectious diseases [1]. These include emerging and re-emerging diseases, like e.g., coronavirus disease 2019 (COVID-19), monkeypox (MPOX), Ebola virus disease (EVD), polio, cholera, Rift Valley fever, Crimean-Congo hemorrhagic fever, and yellow fever[2].

Since the adoption of the International Health Regulations (IHR) 2005, World Health Organization (WHO) Member States (MS) established systems to minimize the effects of PHEs by improving preparedness and response capabilities [3-5]. However, recent PHEs revealed important gaps in IHR core capacities. Previous experiences, particularly the EVD outbreak in West Africa in 2014-2016, revealed critical gaps in preparing for and responding to PHEs. The governments of Guinea, Liberia, and Sierra Leone have acknowledged that poor coordination of efforts and weak health systems hampered the effectiveness of the 2014–2016 EVD outbreak response [6]. Improving preparedness and response capacities of MS as required by IHR (2005) which requires State Parties to develop, strengthen and maintain their capacity to effectively respond to PHEs was one of the recommendations made following this EVD outbreak [6].

A functional Public Health Emergency Operations Center (PHEOC) is a crucial component in meeting the IHR (2005) minimum capabilities and the need to establish a functional PHEOC has been covered as one of the key thematic areas in the Joint External Evaluation (JEE) developed to help MS assess their IHR-related capacities to prevent, detect and respond to public health threats [4]. A PHEOC is a hub for effective coordination of information and resources during the management of PHEs [7, 8]. The PHEOC employs an incident management system (IMS), an emergency management structure with sets of procedures and protocols to provide a coordinated approach for all types of PHEs. However, only after a PHEOC has been thoroughly implemented and continuously strengthened can it serve as an effective platform for managing and coordinating information and resources among the multidisciplinary and multisectoral agencies, governments, organizations, and other stakeholders involved in PHEs preparedness and response.

After the 2014 to 2016 EVD outbreak in West Africa, WHO Regional Office for Africa (WHO AFRO), Africa Centres for Disease Control and Prevention (Africa CDC), West African Health Organization (WAHO), and other partners heightened the support to MS to establish and operationalize PHEOCs as the top priority. As part of the support, WHO published an evidence-based Framework for a Public Health Emergency Operations Center in 2015 [7]. In the same year, WHO AFRO officially launched a regional emergency operations center network (AFR-EOCNET) to collaborate with MS and key partners to assist MS in establishing functional PHEOCs and to promote timely exchange of experiences, best practices, and information [9]. Furthermore, the collaboration between WHO AFRO, Africa CDC, and United States Centers for Disease Control and Prevention (US CDC) since 2018, was instrumental in the development of an easy-to-use handbook for PHEOC operations and management and a PHEOC legal framework guide which provides technical guidance to MS on the development of appropriate policies that authorize PHEOC establishment and full functionality [4, 5].

The PHEOC framework highlights that a fully functional PHEOC is achieved and maintained by putting in place four key components: policies, plans, and procedures; information system and data standard; skilled human resources; and communication technology and physical infrastructure. Africa has made considerable strides in establishing PHEOCs by putting in place these key components to strengthen emergency management. Findings from regional-level meetings on PHEOC implementation and other activities and exchanges, including JEEs, revealed that there are remaining challenges in establishing functional PHEOCs in the continent. Until now, no detailed assessment on the status of PHEOC establishment and functionality in Africa was available. Therefore, WHO in partnership with Africa CDC, WAHO, US CDC, United Kingdom Health Security Agency (UKHSA), Bill and Melinda Gates Foundation (BMGF), and Robert Koch Institute (RKI) conducted a survey to assess the implementation

For peer review only - http://bmjopen.bmj.com/site/about/guidelines.xhtml

status of core components required to having functional PHEOCs in Africa. The main question to be answered by this survey was 'How are the PHEOCs performing in meeting the minimum requirements of the core components for coordinating functional PHE response in the Africa Region'? The specific objectives were to assess the general implementation status of the four PHEOC core components' parameters, and to evaluate the fulfillment status of each core components' minimum requirements.
The survey further intended to answer the following questions

- What are the capacities of the PHEOCs regarding communication technology and physical infrastructure?
- How are the key PHEOC policies, plans, and procedures for emergency management (e.g., legal framework, multi-hazard plan, PHEOC plan/handbook, hazard-specific plans, functional plans, SOPs, etc.) developed and implemented?

What are the PHEOCs capacities in terms of routine and surge emergency management workforce?
The findings of this survey could serve as a resource for planning considerations, generate evidence to contextualize minimum and core requirements for functional PHEOCs, and contribute to policy recommendations.

METHODS

Data collection

A standardized self-assessment tool was developed based on Annex 9 of the PHEOC framework to determine the PHEOC minimum requirements (Checklist for Implementing a PHEOC[7]). It encompasses 31 questions resulting in variables for the analysis across the four defined PHEOC core components to assess the implementation status and existing capacities. Each question allowed for a yes or no answer as well as a comment section to provide additional information if desired. The questionnaire was conceived in Kobo Toolbox and finalised in May 2021, the survey link was shared with WHO country offices and then shared with PHEOC focal points of all countries, and these could complete and submit their responses online. The online survey questionnaire was shared with 54 MS in Africa (47 MS from WHO African Region) and these responded based on their own first-hand experience. Data were collected between May and November 2021. In addition, the Regional Office set up a one-on-one discussion with focal points from selected countries to verify and ensure completeness their responses.
To allow for a structure, systematic approach, and comparison, different parameters were selected under each PHEOC core component (number of variables per component, **Table 1**) and used to assess the operationalization of PHEOCs across the Region.

Table 1. Assessed variables by PHEOC core component in Africa, 2021*

Core components of a PHEOC	*Variables assessed (Number)*
PHEOC policies, plans, and procedures	12
Human resources, training, and simulation exercises	7
Information management and data standards	4
Communication technology and physical infrastructure	8
Total	31

* Results are only presented for selected parameters in the next section

Data analysis

In addition to all variables collected for the general PHEOC core components status analysis, the minimum required PHEOC capabilities for running critical emergency response functions were selected from these variables included in the survey tool. This selection was done based on the WHO framework for a PHEOC and expert consultation. Members of the above-mentioned group of partners committed to strengthening PHEOC capacities in Africa prioritized and selected specific parameters within each core component based on PHEOC operations and consensus. As a result, 19 of the 31 (**Table 2**) parameters from the four PHEOC core components were prioritized and classified the PHEOCs into three categories for planning purpose: PHEOCs meeting 80% and above of the minimum requirements

For peer review only - http://bmjopen.bmj.com/site/about/guidelines.xhtml

(n≥15 of the 19) were labeled as "fully functional", PHEOCs meeting 60%-79% (11-14 of the 19) as "functional", and PHEOCs that met below 60% (≤10 of the 19) labeled as "partially functional". The survey data was extracted from Kobo Toolbox into Excel used to analyze the data. Simple frequencies and proportions were calculated for each of the four core components' variables and the above-mentioned scale used for a general PHEOC classification into one of the three categories regarding their functionality. The same weight was given to each parameter assess PHEOC functionality. For selected variables, additional calculations were made for those MS part of the WHO African Region (subgroup).

Table 2. Parameters used to classify the PHEOC core components minimum requirements fulfillment level in Africa, 2021

PHEOC policy, plans, and procedures	Human resource, training, and simulation exercise	Information management and data standards	Communication technology and physical infrastructure
The legal framework authorizes a PHEOC at the national level	PHEOC has the minimum requirements for routine staff (PHEOC manager and key IMS)	A direct link to the national surveillance structure exists	Dedicated PHEOC facility with adequate workstations for the key IMS functions
Legal frameworks include governance structure, core functions, & authority	Staff trained in PHE preparedness and response	Essential data systematically flow to the PHEOC from relevant sectors	Availability of internet access for all workstations and meeting rooms
Relationships, before, during, and after a PHE between stakeholders defined	PHEOC has a comprehensive exercise program involving more than one exercise per year	Able to collect and manage operational information to inform leadership	Availability of electricity with backup power
A policy group to provide strategic guidance established	Staff are routinely trained on guides, plans, and through simulation to validate competencies	PHEOC uses digital solutions to process its information	
Handbook for PHEOC operations and management is in place	Contact roster of trained personnel to fill IMS positions when PHEOC activated is available		
The multi-hazard response plan is approved			
A business continuity plan (BCP) is in place			

Patient and Public involvement

None.

RESULTS

A total of 51 (93%) MS PHEOC focal points in the African continent responded to the survey. Forty-one (80%) reported that they have an established PHEOC at the national level and 21 of these MS also had a PHEOC at the sub-national level. Forty-one of the 47 WHO African Region MS in the WHO responded and 36 reported existence of a national PHEOC, 17 of these also had one at the sub-national level.

For peer review only - http://bmjopen.bmj.com/site/about/guidelines.xhtml

Table 3 illustrates that 12 (29%) of the 41 national PHEOCs in the continent and 11 (31%) PHEOCs in the WHO African Region met 80% or more of the minimum requirements for the four core components of a PHEOC and classified as fully functional. In addition, twelve (29%) of the PHEOCs in the continent met 60%-79% and 17 (41%) met below 60% the minimum requirements and classified as functional and partially functional, respectively. The number of PHEOCs in the continent that meet the minimum requirements by PHEOC core components are displayed in **Table 3**.

Table 3. PHEOC minimum requirements fulfillment by core component in Africa, 2021

Core components of a PHEOC	*Fully functional: PHEOCs that met ≥80% (`≥15 of 19) # (%)*	*Functional: PHEOCs that met 60%-79% (11-14 of 19) # (%)*	*Partially functional: PHEOCs that met ≤50% (≤10 of 19) # (%)*
PHEOC policies, plans, and procedures	10 (24)	17 (34)	14 (34)
Human resources, training, and simulation exercises	17 (41)	7 (29)	16 (39)
Information management and data standards	24 (59)	10 (24)	7 (17)
Communication technology and physical infrastructure	18 (44)	11 (27)	12 (29)
Total (n=41)	**12 (29)**	**12 (29)**	**17 (41)**

Core component 1: PHEOC policies, plans, and procedures

Twenty-five (61%) MS with PHEOCs have developed and enacted a legal framework to establish it, and 23 (56%) of the PHEOC legal frameworks have covered governance structure, core functions, and the scope of authority (**Figure 1**). In addition, twenty-nine (71%) MS PHEOCs had a policy group to provide strategic guidance for the PHEOC operationalization.

Twenty (49%) MS implemented a handbook for PHEOC operations and management to guide their day-to-day operations. Twenty-five (61%) have developed a national multi-hazard response plan that includes the concept of operations addressing priority risks and 19 (46%) of them were endorsed. In 24 (59%) MS with PHEOCs, a response plan covering various stakeholders' roles and responsibilities supporting response efforts was in place. Thirty-one (76%) of the PHEOCs had an IMS or comparable response structure and 25 (61%) could activate within 120 minutes as per JEE recommendation (**Table 4**).

In the WHO African Region, 23 (56%) MS had a BCP and 18 (44%) had a handbook for PHEOC operations and management. Besides, 17 (41%) and 23 (56%) MS had an approved multi-hazard response plan and the capacity to activate their PHEOC within 120 minutes, respectively.

For peer review only - http://bmjopen.bmj.com/site/about/guidelines.xhtml

Table 4. Selected PHEOC capacities for core component 1 (PHEOC policies, plans, and procedures) in Africa, 2021

PHEOC plans and procedures	*PHEOCs*	
	**Number (#)**	**Proportion (%)**
Operational IMS or comparable structure in place	31	76
Developed multi-hazard response plan incl. the concept of operations	25	61
PHEOC activation possible within 120 minutes after decision	25	61
Available response plan covering roles & responsibilities of organisations supporting the response	24	59
Business Continuity Plan (BCP) in place	23	56
Implemented handbook for PHEOCs operations & management	20	49
Approved multi-hazard response plan incl. concept of operations	19	46

Core component 2: Human resources, training, and simulation exercises

Thirty-eight (93%) of the PHEOCs indicated that at least one manager or focal point was present to oversee day-to-day operations and management of the PHEOCs and 26 (63%) had the minimum expected personnel (PHEOC manager, lead for operations, planning, logistics, finance and administration, communications officer, and ICT expert) to carry out routine preparedness activities. In 17 (41%) PHEOCs, staff were frequently oriented on PHEOC guides, and emergency management, and 14 (34%) PHEOCs regularly simulated exercises (SimEx) to test different capacities. When the IMS was activated, 32 (78%) PHEOCs could contact a roster of pre-trained experts to assist with response coordination activities (**Table 5**).

In the WHO African Region, 21 (51%) PHEOCs had the minimum expected routine staff, and 29 (71%) PHEOCs had staff trained on PHE preparedness and response.

Table 5. Selected PHEOC capacities for core component 2 (human resources and simulation exercises) in Africa, 2021

Human resources, training, and simulation exercises	*PHEOCs*	
	**Number (#)**	**Proportion (%)**
Designated PHEOC Manager	38	93
Staff trained on PHE preparedness and response	33	80
Contact a roster of trained personnel	32	78
Personnel to run response functions 24/7	26	63
*Minimum routine staff for IMS functions**	26	63
Dedicated training program	21	51
Routinely trained staff on existing PHEOC guidance documents and exercises conducted to validate competencies	17	41
More than one exercise program per year and documented after-action reviews	14	34

*PHEOC manager and leads for operations, planning, logistics, finance and administration, communications officer, & ICT manager.

For peer review only - http://bmjopen.bmj.com/site/about/guidelines.xhtml

Core component 3: Information management and data standards

There was a digital solution to process the data and information acquired from various sources in 33 (80%) of the PHEOCs. Data were routinely coming from relevant sectors (e.g., surveillance and/or local health systems) in 32 (78%) PHEOCs. Thirty-five (85%) PHEOCs could collect and manage operational information (situational awareness) to inform action, whereas 26 (63%) PHEOCs were producing visual dashboards to convey a concise picture of the response and situation. In the WHO African Region, 31 (86%) PHEOCs could manage operational information to inform action and data were flowing systematically in 29 (81%) PHEOCs.

Core component 4: Communication technology and physical infrastructure

As indicated in **Figure 2**, 27 (66%) of the PHEOCs had sufficient computer workstations, whereas 33 (80%) had workstations capable of serving the essential IMS personnel. Twenty-six (63%) had internet connectivity for the workstations and meeting rooms and 27 (66%) PHEOCs could hold a teleconference.

Twenty-eight (78%) and 21 (51%) PHEOCs with space for at least the IMS staff and adequate internet access, respectively, were from MS of the WHO African Region.

DISCUSSION

The authors present findings on implementation status of PHEOCs at national levels in Africa from a survey conducted between May and November 2021 with the aim of assessing the progress made in PHEOC establishment and implementation status of the core components that make a PHEOC functional. Besides, it was also driven by the interest to have a resource document to facilitate evidence-based planning and support towards achieving functional PHEOCs in all MS in the African continent.

The findings showed that the majority of the countries in the continent have designated a PHEOC either in a temporary or permanent facility. They further revealed that countries made efforts in implementing the four core components of a PHEOC including developing plans and procedures, train experts on PHEOC operations and IMS, strengthening information management and equipping a PHEOC with ICT infrastructure. However, the implementation level of the core components varies from MS to MS.

According to the results, MS made significant progress as 41 (80%) of them established a PHEOC since the commencement of the regional initiative in 2015 that aimed to support MS with establishing PHEOCs to strengthen their emergency management capacity. This initiative was further intensified following the recommendations from the 2014-2016 EVD outbreak in West Africa and different stakeholders joining the effort to strengthen emergency preparedness and response capabilities of MS [6]. The results, however, showed that only 12 (29%) of the PHEOCs in the Africa met at least 80% of the minimum requirements and are fully functional. Furthermore, 12 (29%) and 17 (41%) of the PHEOCs in the continent met 60%-79% and below 60% the minimum requirements and classified as functional and partially functional, respectively. The capacity related to the basic indicators, however, differed. For example, in most of the MS, the core components of PHEOC policies, plans, and procedures and the PHEOC workforce appear to have a critical gap, where only 10 (24%) and 17 (41%) met 80% and above of the minimum requirements. The main reason for this could be technical capacity, financial or other constraints. Most of the MS, on the other hand, had the required capability in terms of core component 3, Information management, and data standards.

Adverse health outcomes and economic disruption attributed to PHEs requires effective preparedness and response procedures [2,10]. A legal framework for approving the PHEOC's establishment and operation, a handbook for its operations and management, event or hazard-specific response and management plans, an incident action plan, and other pertinent plans and procedures must ideally be in place before any PHE occurs [7]. With respect to the core component of PHEOC policies, plans, and procedures, only 20 PHEOCs had a handbook for PHEOC operations and management and a multi-hazard response plan including the concept of operations. Besides, a business continuity plan (BCP) was in place in only 23 PHEOCs. This might affect the effectiveness of the PHEOC to coordinate response during emergencies due to the absence of clear guidance and operational response plans.

For peer review only - http://bmjopen.bmj.com/site/about/guidelines.xhtml

Regarding the workforce capacity, despite most of the PHEOCs meeting the key requirements for some capabilities, only half of them had a dedicated training program to train routine PHEOC and surge staff. Only 41% of MS PHEOCs had staff routinely trained on existing PHEOC guidance documents and conducted exercises to validate competencies. Besides, 34% of the PHEOCs had more than one exercise program per year and implemented documented after-action reviews to address the gaps identified during exercises. Though the personnel needed for a PHEOC may vary due to many factors, including the emergency's size and complexity, a PHEOC usually has routine staff responsible for daily activities while surge personnel will be mobilized when the PHEOC is activated. The surge personnel, therefore, should be identified and trained in IMS and specific functions prior to PHE and an up-to-date roster should be maintained [11]. When the PHEOC is activated, it should be staffed with a team of subject matter experts drawn from the roster and regular training and exercises should be conducted to ensure the functioning, staffing and availability of a trained and skilled personnel [11-15].

An EOC's lifeblood is information. Information management is a critical component of a functional PHEOC, it entails gathering, analyzing, interpreting, and distributing data promptly [8-12, 16]. Event-specific data, event management information, and context data are the three categories of data that must be consistently recorded, analyzed, interpreted and displayed in a PHEOC [7]. During response coordination, a PHEOC requires specific types of data depending on the type of PHE. Data should be gathered according to local, i.e. context- and event-based conditions. During activation, EOCs use data technologies and informal networks of public health professionals to monitor epidemiological data and field reports from several sources [13]. In addition, the PHEOC should maintain clear and updated information about the incident or disaster. Effective communication is mandatory to retain the public's trust in messages and the function of the PHEOC [17].

The majority of the PHEOCs had better performances for most parameters under the core component of communication technology and physical infrastructure. However, there were critical gaps in having interoperable radio communication and logistic facilities. The primary reason for this might be limitations in internet accessibility, funding, and technical capabilities. This primarily affected the effectiveness of the PHEOC functionality in terms of communication ease, especially at the sub-national level. Furthermore, the findings showed that most of the MS only have PHEOC capacity at the national level and there are important gaps at the sub-national level, where most emergencies are managed. Addressing these gaps should urgently be considered in MS with lower emergency operations' capacity. The PHEOC should have the potential to acquire the technologies such as computers, phones, TV plasma screens, projectors, and radio systems that support telecommunications, information management, and visualization of operational information resulting in more effective response coordination [18].

LIMITATION

The survey was self-administered hence respondents did possibly not appraise performance status on the same scale. The largely binary nature of the questionnaire (yes/no options) may have limited the documentation of minor but important progress that was made. Last but not least, the survey does not deliver in-depth information for more detailed insight, as the main purpose was obtaining an overview of the PHEOC implementation status.

CONCLUSION

WHO Member States in Africa made significant progress in setting up PHEOCs to improve their emergency management capabilities, the majority (80%) established a PHEOC since the regional PHEOC strengthening initiative started in 2015. Of these, one-fifth (12) MS have a national PHEOC fulfilling 80% or more of the minimum requirements to operate critical emergency functions and was classified as fully functional. The remaining have PHEOCs were classified as functional but needing improvement (29%) or partially functional (41%), respectively. PHEOCs in many MS have varying capabilities and need improvement to be fully functional and some MS (10) still have no PHEOCs to coordinate PHE response coordination.

For peer review only - http://bmjopen.bmj.com/site/about/guidelines.xhtml

The main bottlenecks for implementing functional PHEOCs meeting the requirements in all the four core components in Africa include the absence of a legal framework that clearly defines its mandate and functions, the lack of a standing policy group to provide operational support and strategic direction, unapproved plans and procedures, and limited availability of skilled human resources and funding for operations and sustainability.

This is the first in-depth analysis of PHEOC implementation status in Africa; the study was able to determine the implementation status of previously defined key capacities. The findings could be used for planning considerations in order to further improve PHE response capacities on the continent. It is crucial to enhance the implementation of functional PHEOCs according to the WHO framework and accompanying handbooks and guides. The implementation planning and execution processes require the involvement of all relevant stakeholders. Besides, regional, and international partners need to support MS' efforts to address these gaps in developing functional PHEOC.

Contributors

FST, WM, WE, VK, YK, AA, JL, MT, CM, ER, VB, EC, LM, AH, MA, CIN, BF, and GAS prepared the survey tool. ST and AL conducted relevant article searches, developed a data analysis plan, analyzed the data, and wrote the manuscript. FST, AL, WM, WE, VK, YK, AA, JL, MT, CM, ER, VB, EC, LM, AH, MA, CIN, BF, and GAS contributed to the writing and reviewed the final manuscript. All authors read and approved the final manuscript.

Funding statement

This work received no specific grant from any funding agency in the public, commercial or not-for-profit sectors.

Competing interests

All authors declare they have no competing interests with regards to this work.

Ethics Statement

Ethical approval was not required. The data collected in the frame of this study did not involve any personally identifiable information. Respondents invited to fill in the online questionnaire were informed about its purpose, aim and objectives, and were aware that by providing their answers they consented to participating.

Availability of data and materials

The datasets from the survey used in this article are available upon reasonable request.

Acknowledgment

We thank all the MS in the continent for their significant contributions to the survey. We also want to thank everyone who helped review the survey tool and article.

REFERENCES

1. World Health Organization, Standard Operating Procedures for AFRO Strategic Health Operations Center (AFRO SHOC).
2. Talisuna AO, Okiro EA, Yahaya AA, Stephen M, Bonkoungou B, Musa EO, et al. Spatial and temporal distribution of infectious disease epidemics, disasters, and other potential public health emergencies in the World Health Organisation Africa region, 2016-2018. Global Health. 2020;16(1):1–13.
3. WHO. This booklet introduces all 11 sections of the Integrated Disease Surveillance and Response (IDSR) Technical Guidelines. 2019;
4. Kamradt-Scott A. The International Health Regulations (2005). Int Organ Law Rev. 2019;16(2):242–71.
5. WHO. Public health events of initially unknown etiology : A framework for preparedness and response in the African Region. 2014; Available from: http;//www.who.int/iris/handle/10665/112832
6. Agbo S, Gbaguidi L, Biliyar C, Sylla S, Fahnbulleh M, Dogba J, et al. Establishing National Multisectoral Coordination and collaboration mechanisms to prevent, detect, and respond to

For peer review only - http://bmjopen.bmj.com/site/about/guidelines.xhtml

public health threats in Guinea, Liberia, and Sierra Leone 2016–2018. One Heal Outlook. 2019;1(1):1–13.
7. WHO. Framework for a Public Health Emergency Operations Centre. MMWR Morb Mortal Wkly Rep [Internet]. 2015;50(RR13)(July 27):1–35. Available from: www.who.int/about/licensing/%0Ahttps://apps.who.int/iris/bitstream/handle/10665/196135/9789241565134_eng.pdf?sequence=1
8. WHO; A Systematic Review of Public Health Emergency Operations Centres (EOC). 2013;(December):11.
9. Network of Public Health Emergency Operation Centers established to protect lives _ WHO _ Regional Office for Africa.
10. World Health Organization. Strengthening health-system emergency preparedness. 2012;96. Available from: http://www.euro.who.int/__data/assets/pdf_file/0008/157886/e96187.pdf
11. Xu M, Li SX. Analysis of good practice of public health Emergency Operations Centers. Asian Pac J Trop Med [Internet]. 2015;8(8):677–82. Available from: http://dx.doi.org/10.1016/j.apjtm.2015.07.006
12. FEMA. National Incident Management System 2017 learning materials. 2017;
13. Balajee SA, Pasi OG, Etoundi AGM, Rzeszotarski P, Do TT, Hennessee I, et al. Sustainable model for public health emergency operations centers for global settings. Emerg Infect Dis. 2017;23(December): S190–5.
14. CDC. CDC Emergency Operations Center | CDC. CDC Emergency Operations Center | CDC. 2020.
15. Olu O, Usman A, Kalambay K, Anyangwe S, Voyi K, Orach CG, et al. What should the African health workforce know about disasters? Proposed competencies for strengthening public health disaster risk management education in Africa. BMC Med Educ. 2018;18(1).
16. Scanlon J. The Role of EOCs in Emergency Management: A Comparison of American and Canadian Experience. Vol. 12, International Journal of Mass Emergencies and Disasters. 1994. p. 51–75.
17. Rebmann T, Carrico R, English JF. Lessons public health professionals learned from past disasters. Public Health Nurs. 2008;25(4):344–52.
18. Simon R, Teperman S. WTC Lessons for disaster management. 2001;5(6):5–7.

For peer review only

For peer review only - http://bmjopen.bmj.com/site/about/guidelines.xhtml

List of Figures

Figure 1. PHEOCs with the capacity for core component 1 (PHEOC policies, plans, and procedures) in Africa, 2021

Figure 2. PHEOCs with the capacity for core component 4 (communication technology and physical infrastructure) in Africa, 2021

For peer review only

For peer review only - http://bmjopen.bmj.com/site/about/guidelines.xhtml

Figure 1. PHEOCs with the capacity for PHEOC core component 1 (PHEOC policies, plans, and procedures) in Africa, 2021

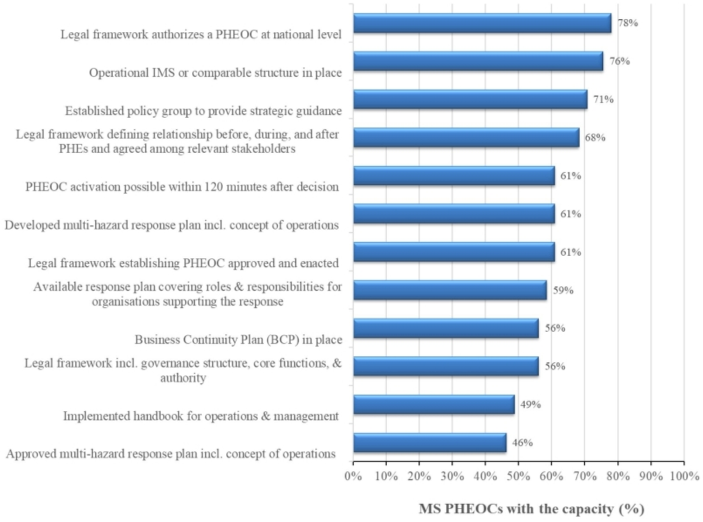

215x279mm (300 x 300 DPI)

Figure 2. PHEOCs with the capacity for core component 4 (communication technology and physical infrastructure) in Africa, 2021

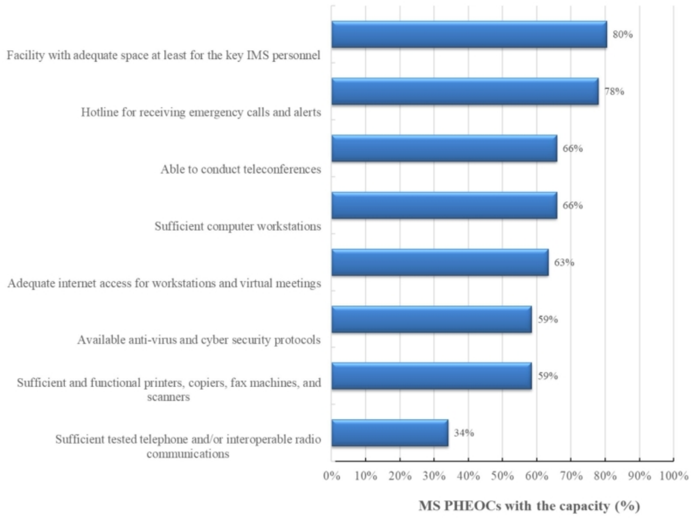

215x279mm (300 x 300 DPI)
